# Historical texts as a potential resource for plant-based antiviral agents against SARS-CoV-2: the example of the Receptarium of Burkhard III von Hallwyl from 16th-century Switzerland

**DOI:** 10.3389/fphar.2025.1731629

**Published:** 2026-02-19

**Authors:** Nina Vahekeni, Jonas Stehlin, Corinna Urmann, Evelyn Wolfram, Yannick Geissmann, Yelena Ruedin, Samuel Peter, Olivier Engler, Andreas Lardos

**Affiliations:** 1 Natural Product Chemistry and Phytopharmacy Group, School of Life Sciences and Facility Management, Institute of Chemistry and Biotechnology, ZHAW Zurich University of Applied Sciences, Wädenswil, Switzerland; 2 Organic-Analytical Chemistry, Weihenstephan-Triesdorf University of Applied Sciences, Straubing, Germany; 3 TUM Campus Straubing for Biotechnology and Sustainability, Technical University of Munich, Straubing, Germany; 4 Spiez Laboratory, Federal Office for Civil Protection, Spiez, Switzerland

**Keywords:** ethnopharmacology, *Geranium robertianum*, medicinal plants, SARS-CoV-2, vero cells E6, *Viola odorata*

## Abstract

**Background:**

In search for effective prophylactic and possibly curative therapeutics against SARS-CoV-2, an historical-ethnopharmacological approach was used to select plants described in the Receptarium of Burkhard III von Hallwyl (RBH), a recipe text from 16th century Switzerland. Ten plant species were selected from the pre-established RBH database based on specific historical uses presumably linked with the treatment of viral infections.

**Methods:**

From each plant aqueous and hydroethanolic extracts were produced. CellTiter-Glo® Luminescent Cell Viability Assay was used to assess antiviral activity against SARS-CoV-2 and the effect on cell viability of the extracts.

**Results:**

Of the ten plants tested, four displayed an antiviral activity ≥ 50% at 16.7 μg/ml with acceptable cell viability (> 75%): *Artemisia vulgaris* L. (aerial parts), *Geranium robertianum* L. (arial parts), *Sambucus nigra* L. (leaves) and *Viola odorata* L. (leaves). The crude extracts were partitioned in aqueous and organic pre-fractions and further analyzed. The ethyl acetate pre-fractions of *G. robertianum, S. nigra*, and *V. odorata* expressed significant antiviral activity of nearly 100% at 5.6 μg/ml (P < 0.05). The most potent inhibitory activity was observed for the ethyl acetate pre-fraction of the leaves of *Viola odorata* with 87% at 1.9 μg/ml (P < 0.0001). Alongside bioactivity testing, phytochemical fingerprints were made, with the aim to provide a preliminary characterization of the active crude extracts. An overview of published phytochemical and antiviral data on the four active plants reveals a fragmentary picture, especially when considering the plant parts investigated. Despite of the promising antiviral effects observed in our study, further in-depth pharmacological and phytochemical investigations are required to comprehensively evaluate the potential of our candidates.

**Conclusions:**

Our study suggests that an ethnopharmacological approach based on historical records of plant use in combination with a rational selection and testing procedure allows to identify interesting candidates, even among medicinal plants no longer in use. The process of selecting plants from RBH also illustrates the challenges associated with the study of historical texts, particularly the interpretation of the medicinal uses and the assessment of the botanical identities of historical plant names.

## 1 Introduction

### 1.1 General background of the study and the Receptarium of Burkhard III von Hallwyl

Respiratory viruses, such as influenza and coronaviruses (CoVs), have repeatedly triggered deadly disease outbreaks. Major flu pandemics include the Spanish flu (Influenza A/H1N1, 1919), Asian flu (Influenza A/H2N2, 1959), and Hong Kong flu (Influenza A/H3N2, 1970) (Piret and Boivin, 2021). Two coronavirus outbreaks left indelible marks: the Severe Acute Respiratory Syndrome coronavirus (SARS-CoV) in 2002/2003 and the Middle East Respiratory Syndrome (MERS-CoV) occurring from 2012 to 2015 (Song et al., 2019). Recently, the Severe Acute Respiratory Syndrome Coronavirus 2 (SARS-CoV-2) pandemic caused more than 7 million deaths globally (WHO Coronavirus (COVID-19) Dashboard, https://covid19.who.int/). As of March 2025, there have been more than 777 million reported cases of COVID-19 (WHO Coronavirus (COVID-19) Dashboard, https://covid19.who.int/). Given the traumatic impact of influenza and coronavirus outbreaks, and specifically, the COVID-19 pandemic, along with the limited availability of effective preventive treatments against SARS-CoV-2 infections and cold-related infections, investigating plants or metabolites as an additional therapeutic intervention may contribute to broadening prophylactic options.

The potent anti-coronavirus activities (COVID-19, SARS-CoV, MERS-CoV) of natural products have been summarized in several reviews (Huang et al., 2020; Verma et al., 2020; Attah et al., 2021; Kim, 2021; Llivisaca-Contreras et al., 2021; Remali and Aizat, 2021; De Oliveira et al., 2022; Khuntia et al., 2022; Latha et al., 2022). Various phytochemicals, including polyphenols, alkaloids, and terpenoids, were identified as potential active anti-SARS-CoV-2 agents (Adhikari et al., 2021). Compared with the large majority of investigations evaluating the in silico potential of plant metabolites, few studies reported in vitro anti-SARS-CoV-2 activity of plant extracts (Nair et al., 2022; Chen et al., 2023; De Araujo et al., 2023; Farooq et al., 2024; Kakimoto et al., 2024; Rodrigues et al., 2024). Certain plants with a long history of use in traditional medicine systems to manage flu syndromes, such as *Withania somnifera* (L.) Dunal, *Andrographis paniculata* (Burm.F) Wall. ex Nees, *Artemisia annua*, or *Nigella sativa* L., have been shown to be promising candidates in the search for an effective preventive or curative treatment for COVID-19 (Nair et al., 2022; Cyril et al., 2023; Ramli et al., 2023; Shanker et al., 2023).

An editorial by Wang et al. (2021) in this journal commented on the research topic “Ethnopharmacological Responses to the Coronavirus Disease,” illustrating the enormous potential of treatment options for this health problem from traditional medicine systems, but also pointing out the challenges faced by research in this context.

In searching for effective agents for the possible development of prophylactic or therapeutic treatment options for illnesses commonly referred to as cold-related, including SARS-CoV-2, we pursue the question of whether traditional knowledge from past eras that has been handed down in writing can offer starting points for research in this regard. In recent decades, the study of historical texts has attracted research interest in ethnopharmacology (for an overview, see Lardos, 2015). More recently, research has been carried out on plants mentioned in historical sources from Europe with the aim of examining them for their different activity profiles (Zimmermann et al., 2012; Wagner et al., 2017; Ulriksen et al., 2022). A notable example illustrating such potential was the development of artemisinin from *Artemisia annua* L. (*Asteraceae*) as a medication for malaria, which was awarded the Nobel Prize in 2015. The discovery goes back to the study of the ancient Chinese recipe text, Emergency Prescriptions Kept Up One’s Sleeve by Ge Hong (284–363 CE) (Hsu, 2006).

Here, we focus on the Receptarium of Burkhard III von Hallwyl (RBH), an early New High German recipe text from Switzerland. The original manuscript is dated to 1580 and comprises 295 folia, or 590 manuscript pages (Frei-Haller, 2005). The manuscript is housed in the State Archives of Canton Bern (Switzerland) in the family archive “von Hallwyl” under the title “Arzneibuch des Burkhard v. Hallwyl, 1580 Original des Verfassers” and with the document signature FA von Hallwyl A 814 (https://www.sta.be.ch/de/start/ueber-uns/staatsarchiv.html). Burkhard III von Hallwyl (1535–1598), the author of the original manuscript, was the lord of Hallwyl Castle, which is located on Lake Hallwyl in the Canton of Aargau, in the German-speaking part of Switzerland. Burkhard III’s real passion was herbal medicine, which he researched in the castle’s own laboratory. He also appears to have treated patients onsite, as suggested by information in the RBH (Frei-Haller, 2005).

However, little is known about the origin of the medicinal knowledge contained in the RBH. While some recipes may have been created by Burkhard III himself, the majority of them appear to have come from people close to him or were adopted from contemporary works with medical content (Frei Haller, 2005; Fankhauser, 2012). The connection of the RBH to other herbal books from that period, which may have served as a basis of knowledge for the recipes recorded in it, is difficult to determine, as there are no corresponding references in the text. However, it can be assumed that the knowledge about the medicinal use of plants conveyed in the RBH shows parallels with other contemporary works. Considering the cultural–historical context, possible resources are German herbals, such as those by Otto Brunfels (1488–1534)—Contrafayt Kreüterbůch from 1537, Leonhard Fuchs (1501–1566)—New Kreüterbuch from 1543, Hieronymus Bock (1498–1554)—New Kreütter Bůch from 1546, or Adam Lonitzer (1528–1586)—Kreuterbůch, New zůgericht from 1557 (Leonti and Verpoorte, 2017; Schmid, 1939).

The RBH belongs to the genre of “Hausarzneibücher,” whose target audience consisted primarily of medical laypeople (Fankhauser, 2012). This is also evident from the text’s preface, which mentions that the work is dedicated to the “common man.” The fact that up to the 18th century, altogether 15 (so far known) handwritten specimens of the RBH were produced and distributed in various regions of Switzerland suggests that the text must have had a particular local significance (Frei, 2012; Frei Haller, 2005). As with other representatives of this genre, the content is primarily a collection of recipes for the medicinal use of plants or other natural substances. The type of knowledge contained in the RBH is comparable to that of other recipe texts from European traditions (Zipser et al., 2023; Oberhelman, 2020): they can be considered distillates of contemporary and earlier knowledge that was considered useful based on empirical criteria and within the respective cultural and historical context.

In a recent study (Stehlin et al., 2024), we explored the dermatological recipes contained in the RBH from a botanical and medicinal point of view and cataloged the data in the RBH database. This relational database comprises 196 recipes that describe the use of 156 plants for 52 different medicinal uses. The term “dermatological recipes” as used there generally refers to recipes in the RBH that include the treatment of externally (on the skin) visible symptoms, regardless of the etiology. Thus, the preparations described in the recipes are not only directed at the treatment of typical skin diseases, wounds, or injuries but also external manifestations of systemic (infectious) diseases. Some of the recipes even describe the preparation of orally applied remedies for the treatment of topically manifest symptoms, such as the Hallwyl wound potion (Stehlin et al., 2024), which was transmitted not only as part of complete manuscript copies, but also as a single item (Barbara Frei Haller, personal communication, 16 October 2024).

### 1.2 Data basis for selecting the plants

To select plants from the RBH for investigation, we used plant use records from the RBH database by Stehlin et al. (2024) that were reported to relate to diseases of viral origin, as one possible interpretation of the historical use in question. A plant use record is defined as the mention of a plant for a particular medicinal use in the RBH database.

To date, only the dermatological recipes contained in the RBH have been elucidated and recorded in the RBH database (Stehlin et al., 2024). As a result, the present study is limited to plants mentioned in this category of recipes that appear to have been used for the treatment of virus-related symptoms on the skin. Consequently, the plants were selected based on their putative pharmacological property (antiviral) and not on the organ system to be treated (e.g., the respiratory tract, which would be obvious in the case of COVID-19). Thus, we argue that plants used to treat skin symptoms of presumed viral origin are generally of interest in the search for natural antiviral agents, regardless of whether there is a direct link between the historical use and the disease in question. Even in the case of various prominent natural products discovered through the study of traditional knowledge, the indications of the drugs developed from them are not related to the traditional use of the source plant. Examples include digitoxin from *Digitalis purpurea* L., galantamine from *Galanthus* species, or vincristine from *Catharanthus roseus* (L.) G.Don (Heinrich, 2010). Finally, there are also many antiviral substances that show a broad antiviral effect across different virus families, such as polymerase inhibitors (e.g., ribavirin, remdesivir, and favipiravir), protease inhibitors (lopinavir), methyltransferase inhibitors, or fusion inhibitors (umifenovir). Because of their multicomponent character, herbal preparations can be expected to have a broad antiviral effect across different virus families. Therefore, the investigation of plants used for virus-related skin symptoms for their activity against other virus families has the potential to identify broadly effective plant-based agents.

As part of our search for potential plant-based therapeutic interventions against cold-related viral infections, this study aims to assess the possible antiviral activities of plants from the RBH database against SARS-CoV-2.

## 2 Materials and methods

### 2.1 Plant selection

The plant species investigated for possible antiviral in vitro activities were selected from plant use records of the RBH database (Stehlin et al., 2024), based on the following criteria: i) At least one of the interpretations of the historical use mentioned in the use record leads to a disease of viral origin, as indicated by Stehlin et al. (2024) (Table 1). The connection between a particular historical use and diseases of viral origin, as reported in that study, is explained in detail using the literature cited below. ii) The RBH plant name mentioned in the use record can be mapped to one specific botanical taxon (i.e., the candidate species for a historical plant name) with a high degree of confidence as the correct identification. For this purpose, we relied on the list of candidate species in Stehlin et al. (2024) (Table 2) and selected only the one candidate species of a given RBH plant name, which was assigned a score of “1” (for details on the procedure for assessing botanical identities followed by Stehlin et al., 2024, see Section 4).

**TABLE 1:** Plants selected for investigation and their historical uses with associated viral diseases.

Plant species [family]^a^	RBH plant name^b^	RBH plant part^c^	RBH Preparation^d^	RBH use^e^	Viral disease^f^
*Alchemilla xanthochlora* agg. (incl. *A. vulgaris* L.) [Rosaceae]	vnnserr frouwen mentle, sinauw	Aerial parts	Decoction in animal fat; Distillate (‘burnt water’)	figwertzenn	Genital warts (Condylomata acuminata)
*Artemisia vulgaris* L. [Asteraceae]	rot bugglenn	Tips of shoot	Decoction in olive oil	figwertzenn	Genital warts (Condylomata acuminata)
*Geranium robertianum* L. [Geraniaceae]	godz gnad	Not mentioned	Distillate (‘burnt water’); Heated bag with the fresh herb	figwertzenn	Genital warts (Condylomata acuminata)
*Plantago lanceolata* L. [Plantaginaceae]	spitzen wägrich	Leaves	Juice of the fresh herb mixed with honey and butter	wolff	Herpes zoster
*Quercus robur* L. [Fagaceae]	eichen	Leaves	Cataplasm of the fresh herb	figwertzenn	Genital warts (Condylomata acuminata)
*Salvia officinalis* L. [Lamiaceae]	edle salbinen	Not mentioned	Decoction in olive oil	figwertzenn	Genital warts (Condylomata acuminata)
*Sambucus nigra* L. [Viburnaceae]	holder	Leaves	Juice of the fresh herb	blattern	Herpes zoster, chickenpox, smallpox
*Stellaria media* (L.) vill. [Caryophyllaceae]	voglj krutt	Not mentioned	Decoction in animal fat	figwertzenn	Genital warts (Condylomata acuminata)
*Veronica officinalis* L. [Plantaginaceae]	eerenpriss	Not mentioned	Distillate (‘burnt water’)	zitter mall	Herpes zoster
*Viola odorata* L. [Violaceae]	viöl, viönlin, blauw viönlin	Aerial parts	Decoction in water; Macerate in olive oil	figwertzenn	Genital warts (Condylomata acuminata)
	vigell	Not mentioned	Macerate in olive oil	blattern	Herpes zoster, chickenpox, smallpox

Data in columns *a* to *e* was adopted from the use record(s) of the respective RBH plant, as listed in Supplementary Material Table S3.

^a^ The plant species selected corresponds to the candidate species for the RBH plant name in question that exhibited a high chance of being the correct botanical identification (score of “1”) (see Supplementary Material Table S3).

^b^ Plant name reported in RBH.

^c^ Plant part used reported in RBH.

^d^ Preparation of the herbal remedy reported in RBH.

^e^ Use of the herbal remedy reported in RBH.

^f^ Diseases of viral origin that are mentioned among the different interpretations of the RBH use in question (see Table 2).

**TABLE 2:** RBH uses with interpretations leading to diseases of viral origin.

RBH use^a^	Interpreting the use in the RBH^b^	Viral disease^c^	Etiology^d^
Blattern	Höfler (1970: p. 49–53): The term is associated with any type of skin rash with blisters or pustules, including boils (furuncles), warts, or Condylomata (see above), and, in particular, also herpes zoster and smallpox (variola) Riecke (2004: Vol. 2, p. 295): “Blatera” are blisters, pustules, or small ulcers (Blatter, Pustel, Hautbläschen, Brandbläschen, kleines Geschwür) Schweizerdeutsches Idiotikon (2023: 5, 208): Blisters or pustules on the skin of different etiologies, including infectious diseases such as smallpox or chickenpox	Herpes zoster Chickenpox Smallpox	Herpes zoster (shingles) and chickenpox are caused by the varicella-zoster virus (VZV), and smallpox is caused by a variola virus of the genus *Orthopoxvirus*.
Schwarz blattern	Höfler (1970: p. 52): A symptom of “blattern” referring to the drying stage of smallpox (variola), the hemorrhagic type of smallpox (variola hemorrhagica), or the blackish blisters of anthrax Riecke (2004): Not found Schweizerisches Idiotikon (2023): Not found	Smallpox	All types of smallpox in humans are caused by a variola virus of the genus *Orthopoxvirus*.
Figwertzenn	Höfler (1970: p. 127): “Feig, fig, gefig, feigwarzen” are primarily genital warts (Condyloma acuminata), while syphilitic warts (Condyloma lata) should also be taken into consideration from the 15th century onward, along with hemorrhoids. Less common uses of the terms include various types of eczema and fleshy outgrowths or neoplasia in different body parts. Riecke (2004: Vol. 2, p. 319): “fig” (feigwarze, kondylom) are wart-like skin proliferations of viral origin Schweizerdeutsches Idiotikon (2023: 16, 1712): “figwertzenn” is a type of wart that usually appears in the genital or anal area, as a manifestation of a venereal disease	Genital warts (Condyloma acuminata)	Genital warts (Condyloma acuminata) are of viral origin and caused by certain types of human papillomavirus (HPV).
Wolff	Höfler (1970: p. 812): Different skin conditions are associated with the disease term: 1) spreading ulceration of the skin (lupus) or on the genitals including moist genital warts (see “figwertzenn”) with sore, burning skin (erythema intertrigo); 2) sore, inflamed, biting skin area (erythema intertrigo) on the anus, buttocks, or groin. 3) any circular, painful skin process such as herpes zoster or specific types of leprosy; 4) bone cancer Riecke (2004): Not found Schweizerdeutsches Idiotikon (2023: 15, 1,556): disease term for various ulcers and spreading rashes	Herpes zoster	Herpes zoster (shingles) is caused by the varicella-zoster virus (VZV).
Zitter mall	Höfler (1970: p. 390): “Zitter mal” are symptoms caused by the disease “zitteroch” (p. 856), which is associated with different manifestations on the skin, including a dry itching skin rash sometimes accompanied by swollen lymph nodes, a skin disorder leaving unpigmented or red skin areas as known in psoriasis, lupus, syphilis, or a poorly healing progressive lichenoid skin rash, along with herpes zoster. Riecke (2004): Not found Schweizerdeutsches Idiotikon (2023: 4, 151): Non-specified temporary manifestations on the skin	Herpes zoster	Herpes zoster (shingles) is caused by the varicella-zoster virus (VZV).

^a^ RBH use: Historical use mentioned in the RBH, adopted from Stehlin et al. (2024): Table 1.

^b^ Interpretations of the historical use were gathered from the literature on premodern and folk terms of medicine with the appropriate cultural and historical background: Höfler’s (1970) Deutsches Krankheitsnamen Buch; Riecke’s (2004) Wörterbuch (Volume 2) of Die Frühgeschichte der mittelalterlichen medizinischen Fachsprache im Deutschen; Schweizerdeutsches Idiotikon for specific terms of the Swiss–German idiom (Schweizerisches Idiotikon, 2023). Interpretations of the historical uses leading to diseases of viral origin (based on the information in Kang et al., 2019, see the column “Viral disease”) are underlined.

^c^ Diseases of viral origin, according to Fitzpatrick’s Dermatology (Kang et al., 2019), are mentioned among the interpretations of the RBH use (see column b).

^d^ Viral etiology of the disease in question, according to Fitzpatrick’s Dermatology (Kang et al., 2019).

To elucidate the historical uses, we relied on the same references as Stehlin et al. (2024): Literature on premodern and folk medicine terms with the appropriate cultural and historical background, including Höfler’s (1970) “Deutsches Krankheitsnamen Buch,” Riecke’s (2004) lexicon (volume 2) of “Die Frühgeschichte der mittelalterlichen medizinischen Fachsprache im Deutschen,” and “Schweizerdeutsches Idiotikon” for specific Swiss–German idiom terms (Schweizerisches Idiotikon, 2023), along with Fitzpatrick’s Dermatology (Kang et al., 2019) as a modern clinical reference to clarify the pathologies of the diseases mentioned.

The procedure is illustrated by the example of the use record of the RBH plant “vnnserr frouwen mentle” for its use in “figwertzenn,” which is one of the plants in our sample. As indicated by Stehlin et al. (2024) (Table 1), the various interpretations of the historical use of “figwertzenn” include genital warts (Condyloma acuminata), a disease of viral origin. Consultation of the relevant source literature to elucidate the viral disease background of “figwertzenn” yielded genital warts and perhaps also syphilitic warts as the most plausible interpretations. Less probable interpretations include hemorrhoids, various types of eczema, and fleshy outgrowths or neoplasms in different body parts (Höfler, 1970: p. 127; Riecke, 2004: vol. 2, p. 319; Schweizerisches Idiotikon, 2023: 16, 1712). According to Kang et al. (2019), genital warts (Condylomata acuminata) are of viral origin and caused by certain types of the human papillomavirus (HPV), whereas syphilitic warts (Condyloma lata) are of bacterial (spirochete) origin and therefore not relevant to our study.

According to Stehlin et al. (2024) (Table 2), *Alchemilla xanthochlora* agg. (incl. *A. vulgaris* L.) is the only botanical attribution available for the RBH plant name “vnnserr frouwen mentle” and is highly likely to be the correct identification (score of “1”). Therefore, *Alchemilla xanthochlora*, with its potential association to a disease of viral origin, is a valid candidate for investigation in the present study.

### 2.2 Sample preparation

Dried plant material was purchased from Dixa AG, Switzerland (see Supplementary Table S1). As far as possible, the same plant parts as mentioned in the respective RBH plant use record were sourced from the supplier. Using standard extraction solvents in phytopharmacy (water and ethanol), two different types of extraction procedures were carried out with the powdered dry plant material. Aqueous crude extracts were prepared by adding a 20-fold quantity of water in relation to the plant material and boiling for 15 min. The decoction was filtered with a Büchner funnel under vacuum or with filter paper. The filtrate was then freeze-dried and stored at −20 °C until use. Hydroalcoholic crude extracts were prepared by adding a 10-fold quantity of 80% (w/w) ethanol in relation to the plant material and extracted at room temperature for 2 h under constant agitation. The extracts were filtered through filter paper, concentrated on a rotavapor (Büchi, Switzerland) at 40 °C until 60 mbar, freeze-dried, and stored at −20 °C until use.

Preselected crude extracts (200 mg each) were partitioned by liquid–liquid extraction using water and ethyl acetate (1:1 v/v) in a separating funnel, allowing for the recovery of two pre-fractions, the aqueous pre-fraction (APF) and the ethyl acetate pre-fraction (EAPF). The chemicals and consumables used are listed in Supplementary Table S2.

### 2.3 Antiviral and cell viability testing

Twenty-two crude extracts (11 aqueous and 11 hydroethanolic extracts) were tested for anti-SARS-CoV-2 activity. Antiviral activity (inhibition of the virus-mediated cytopathic effect) and cell viability (absence of a toxic effect) were assessed on Vero E6 cells. The crude extracts were tested in duplicate against SARS-CoV-2 using the CellTiter-Glo® Luminescent Cell Viability Assay. The antiviral activity of the active extracts was confirmed by a second independent replicate. Crude extracts exhibiting an inhibitory activity ≥50% at a final concentration of 16.7 μg/mL with acceptable cell viability (>75%) were retained for the subsequent pre-fractionation step and underwent partition chromatography. The inhibitory activity and cell viability of the obtained pre-fractions (aqueous pre-fraction (APF) and ethyl acetate pre-fraction (EAPF)) were assessed in the same way as the crude extracts.

#### 2.3.1 Cells and pathogenic viruses

Vero E6/TMPRSS2 cells were passaged in Dulbecco’s modified Eagle medium (DMEM) containing 10% fetal calf serum (FCS), 100 U/mL of penicillin, 100 μg/mL of streptomycin, and 1 mg/mL geneticin (all from Bioswisstec). SARS-CoV-2 (2019-nCoV/IDF0372/2020) clade 19A was obtained from the National Reference Centre for Respiratory Viruses hosted at the Institut Pasteur (Paris, France), propagated in Vero E6/TMPRSS2 cells in DMEM containing 2% FCS, supplements (2%-FCS-DMEM), and 1 mg/mL geneticin at 37 °C, >85% humidity, and 5% CO_2_. The viral titer was determined using a standard plaque assay by incubating ten-fold serial dilutions of the virus for 1 h at 37 °C on a confluent 24-well plate with Vero E6/TMPRSS2 cells. Then, the inoculum was removed, and 1 mL of overlay medium (20 mL DMEM, 5 mL FCS, 100 U/mL of penicillin, 100 μg/mL of streptomycin, and 25 mL of Avicel rc581) was added. After 3 days of incubation at 37 °C, the overlay was removed, and the plates were stained with crystal violet. Due to the clearer readout, Vero E6 cells in suspension were used for the antiviral assays. Vero E6 cells were passaged in minimal essential medium (MEM) containing 10% FCS and supplements (2 mM L-glutamine, non-essential amino acids, 100 U/mL of penicillin, 100 μg/mL of streptomycin, and 1.5 g/L sodium bicarbonate, all from Bioswisstec) at 37 °C, >85% humidity, and 5% CO_2_.

#### 2.3.2 Antiviral assay

The inhibitory activity of the extracts was assessed in a virus pre-incubation experiment. This format allows the extracts to interact directly with and neutralize the virus particles before they can attach to or enter the host cells.

The lyophilized plant extracts were resuspended in DMSO or H_2_O to a concentration of 25 mg/mL and diluted to concentrations of 400 µg/mL, 200 µg/mL, 66.6 µg/mL, 22.2 µg/mL, and 7.4 μg/mL in MEM containing 2% FCS and supplements (2 mM L-glutamine, non-essential amino acids, 100 U/mL of penicillin, 100 μg/mL of streptomycin, and 1.5 g/L sodium bicarbonate, all from Bioswisstec) (2%-FCS-MEM). The plant extracts, 50 µL of each concentration, were distributed in duplicates in the upper half of a flat-bottom, 96-well plate (TPP, Trasadingen, Switzerland) to assess antiviral activity, and 50 µL of the same concentrations of the plant extracts were distributed in duplicate in the lower half of the 96-well plate to assess cell viability or toxicity of the extracts. On each plate, 10 wells were used to determine the maximum viral cytopathogenicity (virus control VC) of infected but untreated cells, and 10 wells were used to determine the maximum cell viability (cell control CC) of uninfected and untreated cells. Further controls included serial dilutions of remdesivir as a positive control and DMSO at the same concentration as the extracts to control for the effects of the diluent. The plates were transferred to the BSL-3 laboratory, and 100 PFU of SARS-CoV-2 virus (2019-nCoV/IDF0372/2020) in 50 µL culture medium (2%-FCS-DMEM) was added to the upper half of the plate and to the virus control (VC) wells, while in the lower half of the 96-well plate, 50 µL of culture medium (2%-FCS-MEM) was added to the cell control wells. The plates were then incubated for 1 h at 37 °C and 5% CO_2_, and 100 µL of Vero E6 cell suspension (2 × 10e^5^/mL) in cell culture medium (2%-FCS-MEM) was added to each well, leading to a final concentration of extracts of 100 μg/mL, 50 μg/mL, 16.7 μg/mL, 5.6 μg/mL, and 1.9 μg/mL. The plates were then incubated for 72 h at 37 °C and 5% CO_2_, after which cell viability was determined by the CellTiter-Glo® Luminescent Cell Viability Assay (Promega, Madison, United States). Briefly, the CellTiter-Glo® suspension was prepared according to the manufacturer’s protocol and mixed 1:1 with the cell culture medium (2%-FCS-MEM. The cell culture medium from the incubated plates was removed, and 200 µL of CellTiter-Glo®/medium mix was added to each well. The plates were shaken for 2 min at 400 rpm and incubated for 10 min at room temperature, and 100 µL of the CellTiter-Glo mix was transferred to a 96-well half-area white flat-bottom plate (Corning® Costar® 3,693, Fisher Scientific).

Luminescence, serving as a surrogate measure of host cell viability by determining cellular ATP levels, was quantified using the GloMax instrument (Promega, Madison, United States). Measurements were taken for the cell control (CC), the virus control (VC), and the experimental wells containing the virus and varying concentrations of the plant extracts (EX). The resulting percent inhibition of the cytopathic effect (CPE) was calculated as detailed in Section 2.5 (Data Analysis).

### 2.4 UHPLC-MS chemoprofiling

Considering best practice recommendations for conducting and reporting the phytochemical analyses of plant extracts used in pharmacological research, the chemical characterization was carried out by following the requirements for “Extract type C,” as described by Heinrich et al. (2022): “Single chemical fingerprinting, each with one or more detection parameters”); optionally, descriptions of marker substances can also be provided. Active crude extracts were characterized using ultra-high-performance liquid chromatography–ultra-violet–mass spectroscopy (UHPLC-UV-MS) fingerprinting at five different detection wavelengths (254 nm, 280 nm, 325 nm, 350 nm, and 410 nm).

The crude extracts were dissolved in ethanol (analytical grade, VWR Chemicals, France) at a concentration of 5 mg/mL, and 2 µL of extracts were injected into an ACQUITY™ Classic UHPLC System consisting of a sample manager, column manager, binary solvent manager, isocratic solvent manager, PDA eλ UV/VIS detector, and quantitative Da (QDA, single quadrupole) MS detector (all, Waters, United States). The chromatographic separation was carried out on a reversed-phase column (ACQUITY HSS T3, 1.8 µm, 2.1 × 100 mm, Waters, United States). The mobile phases A (aqueous formic acid 0.1% V/V) and B (acetonitrile) were used with a flow rate of 0.4 mL/min and a column temperature of 30 °C. Separation was achieved using the following gradient (min/%B): 0/1, 0.2/1,32/99, 35/99, 36/1, and 40/1. The system’s gradient delay volume was 0.14 mL.

After the column passage, the flow was split between the UV and MS detectors at a ratio of 90:10. The flow directed to the MS detector was combined with 0.2 mL/min of make-up solvent, consisting of aqueous acetonitrile 50% V/V, acidified with formic acid 0.05% V/V. UV spectra were recorded between 210 nm and 800 nm at 10 Hz. MS data were collected in positive and negative ion mode (ESI, scan from m/z 100 to 1,250, cone voltage ±15 V, capillary voltage ±0.8 kV, 8 Hz). Empower 3 FR4 (Waters, United States) chromatographic data software was used for data collection and analysis.

Three reference compounds: chlorogenic acid, kaempferol, and violanthin (purchased from EDQM, France, or PhytoLab GmbH and Co. KG, Germany) were used to compare retention times (RTs), UV/VIS spectra, and mass spectra.

### 2.5 Data analysis

Percent cytopathic effect (CPE) inhibition and percent cell viability were calculated as described by Severson et al. (2007). Percent CPE was calculated from the luminescence signal of [(plant extract − virus control)/(cell control − virus control)] × 100. Percent cell viability was defined as the luminescence signal at (plant extract/cell control) × 100 at the final concentration of the extract.

The means of the inhibitory activity of all samples were compared to the relevant solvent control at each respective dosage using a two-way ANOVA, with α = 0.05. Multiple comparisons were corrected using Dunnett’s method. All statistical testing was carried out using GraphPad Prism version 10.1.2 for Windows (GraphPad Software, Boston, Massachusetts, United States).

## 3 Results

### 3.1 RBH plants selected for the study

Ten plant species from nine different botanical families were selected to investigate their *in vitro* antiviral potential (Table 1) following the procedure described in Section 2.1. The selection is based on plant use records from the RBH database (Stehlin et al., 2024) that are associated with historical uses, whose interpretations involve diseases of viral origin. Altogether, 31 corresponding plant use records could be identified in the RBH database (Supplementary Table S3). The historical uses in question are “blattern,” “figwertzenn,” “schwartz blatteren,” “wolf,” and “zitter mall,” which can be roughly translated as blisters, fig warts, black blisters, wolf (lupus), and scattering sign (see Section 3.1.1; Table 2). However, in 15 of the 31 use records, either the plant species was not commercially available or the botanical identity was uncertain. This was because the RBH plant name could not be mapped to one specific botanical species with highconfidence, but it did lead to one or more candidate species that were rated as less plausible. The other 16 use records involve 10 different candidate species with appropriate certainty regarding the botanical identity (score of “1”) that can be associated with four of the above-cited five historical uses: “blattern,” “figwertzenn,” “wolf,” and “zitter mall” (Table 1).

#### 3.1.1 Historical uses in the RBH associated with viral diseases

Five different historical uses are mentioned in the RBH whose interpretations include diseases of viral origin (Table 2).

1. “blattern”: An Ill-defined skin rash or skin disease with blisters, pustules, boils, or warts. Many different illnesses can cause the skin symptoms mentioned, including viral diseases such as herpes zoster (shingles), chickenpox, or smallpox.
2. “schwartz blattern:” The interpretations given point either to different types or symptoms of smallpox, or to anthrax, which is of bacterial origin.
3. “figwertzenn:” The term is primarily associated with condylomas, in particular with genital warts (Condyloma acuminata) of viral origin, but syphilitic warts (Condyloma lata) of bacterial (spirochete) origin must also be taken into account. Hemorrhoids, neoplastic skin changes, swelling, or eczema on the anus are less plausible interpretations that are not related to viral diseases.
4. “wolff:” Different skin conditions are associated with the disease term, especially ulcers, skin inflammations, and spreading rashes, which also include herpes zoster (shingles).
5. “zitter mall:” Different types of skin disorders or manifestations of systemic or infectious diseases on the skin, including also herpes zoster.

Herpes zoster (shingles) and chickenpox are caused by the varicella-zoster virus (VZV), and smallpox is caused by the variola virus of the genus *Orthopoxvirus*. Genital warts (Condyloma acuminata) are caused by certain types of human papillomavirus (HPV).

### 3.2 Anti-SARS-CoV-2 activity

#### 3.2.1 Crude extracts

**FIGURE 1:**
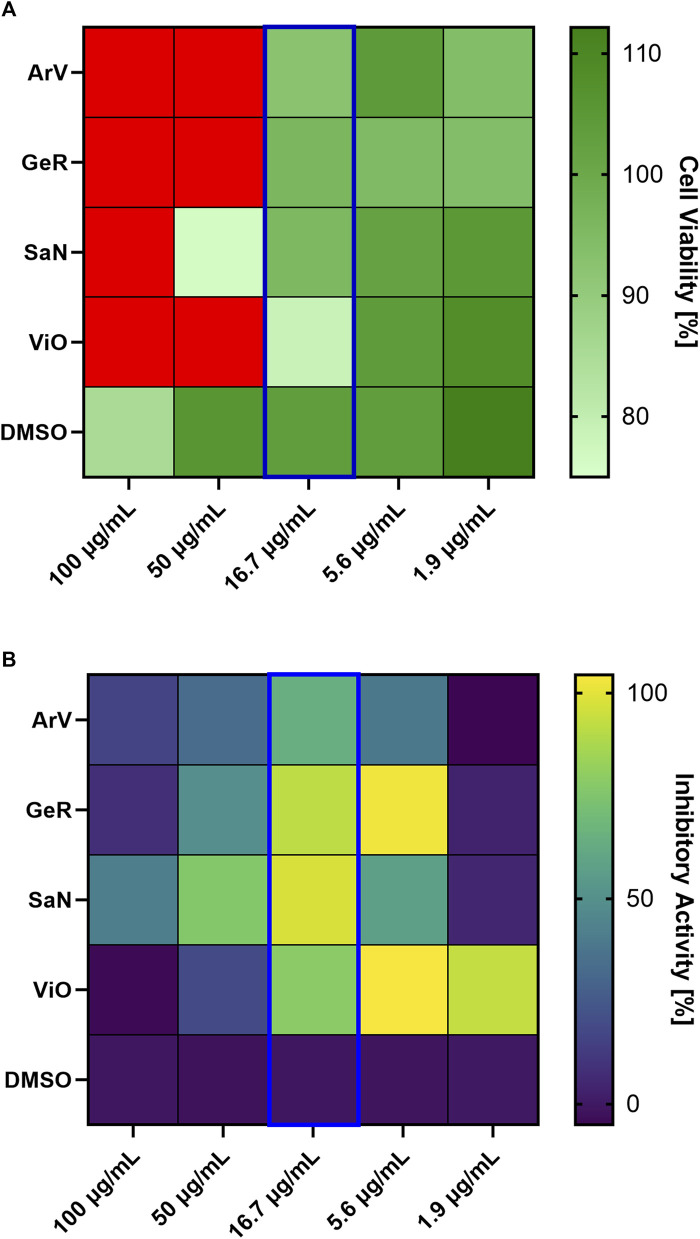
Inhibitory activity **(A)** and cell viability **(B)** of selected crude extracts. The four hydroethanolic crude extracts demonstrating an inhibitory activity of ≥50% at a final concentration of 16.7 μg/mL and cell viability >75% are represented: GeR—*Geranium robertianum*, ViO—*Viola odorata*, ArV—*Artemisia vulgaris*, and SaN—*Sambucus nigra*. **(A)** Inhibition of the cytopathic effect (CPE) at the concentrations tested, expressed as a percentage. The inhibitory activities at 16.7 μg/mL: ArV 65%, ViO 78%, GeR 92%, and SaN 97%. Data represent the means of two independent replicates (n = 2). Negative control—DMSO. Positive control: remdesivir (not shown), with an inhibitory activity of 110% at the tested concentration of 10 μM, and a cell viability of 112%. **(B)** Cell viability at the concentrations tested, expressed as a percentage. Green shades indicate cell viability ≥75%. Red color indicates cell viability <75%.

A total of 22 crude extracts were prepared from different parts of the 10 plant species selected (Table 1) using either water or 80% (w/w) ethanol as extraction solvents. In cases where the plant part to be used according to the RBH (see Table 1) was not specified, the plant part commonly used in herbal medicine was obtained. For *Viola odorata*, two different plant parts (flowers and leaves) were obtained (see Supplementary Table S1). The specification of the extract samples can be found in Supplementary Table S4. All extracts were tested for anti-SARS-CoV-2 activity. Antiviral activity and cell viability were assessed on Vero E6 cells in a serially diluted manner: 100 μg/mL, 50 μg/mL, 16.7 μg/mL, 5.6 μg/mL, and 1.9 μg/mL. Of the 22 crude extracts tested, only the hydroethanolic extracts showed an inhibitory activity (Supplementary Table S5). Four active hydroethanolic extracts, namely *Artemisia vulgaris* (ArV, aerial part), *Geranium robertianum* (GeR, aerial part), *Sambucus nigra* (SaN, leaves), and *Viola odorata* (ViO, leaves), all displaying an inhibitory activity ≥50% at a final concentration of 16.7 μg/mL, were retained for further investigation (Figure 1A). At this concentration point, all four crude extracts showed cell viability >75% (Figure 1B).

#### 3.2.2 Active organic pre-fractions

**FIGURE 2:**
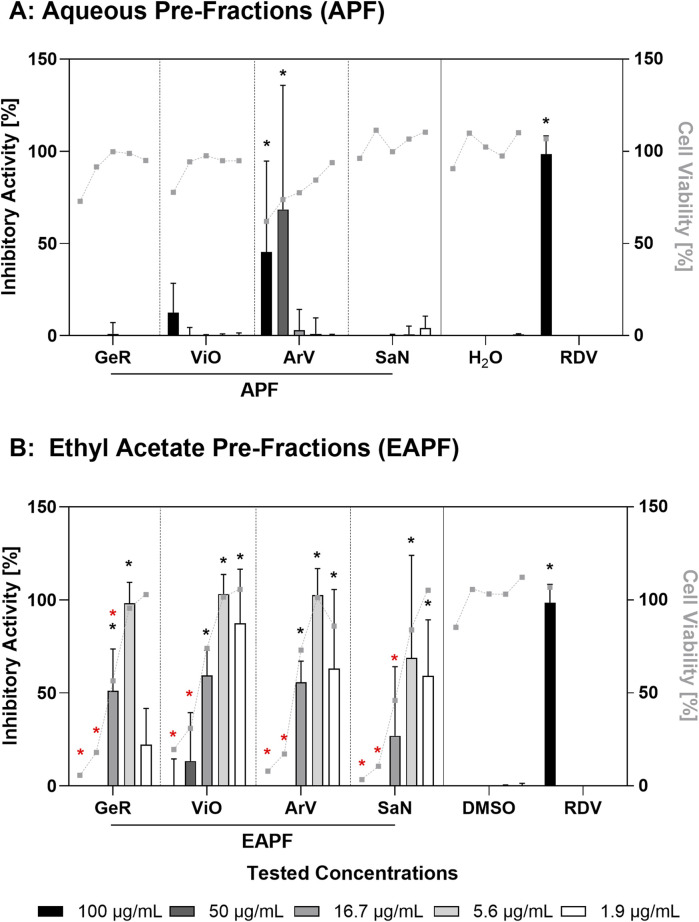
Inhibitory activity and cell viability of the ethyl acetate pre-fractions. Inhibition of the virus-mediated cytopathic effect (bars) and the absence of a toxic effect (gray dots) of **(A)** the aqueous pre-fraction (APF) and **(B)** the ethyl acetate pre-fraction (EAPF). Abbreviations: GeR—*Geranium robertianum*, ViO—*Viola odorata*, ArV—*Artemisia vulgaris*, and SaN—*Sambucus nigra*; H_2_O—solvent control aqueous pre-fractions, DMSO—solvent control ethyl acetate pre-fractions, and RDV—remdesivir (positive control). The means (n = 3) of the inhibitory activity and cell viability at the individual concentrations were compared to their respective solvent controls using a two-way ANOVA with multiple comparisons corrected using the Dunnett’s method. Black asterisks (*) indicate significant inhibitory activity compared to the control, and red asterisks denote the same for cell viability. the dashed lines connecting the cell viability measuring points (gray dots) were added to improve legibility and do not signify continuous measurements.

The four active hydroethanolic extracts from *A. vulgaris* (aerial parts), *V. odorata* (leaves), *G. robertianum* (aerial parts), and *S. nigra* (leaves) were partitioned into organic and aqueous pre-fractions via liquid-liquid extraction. Among aqueous pre-fractions, *A. vulgaris* (ArV/APF) was the only one exhibiting notable inhibitory activity at 50 μg/mL, but this concentration nearly overlapped with the concentration impairing cell viability (Figure 2A). The ethyl acetate pre-fractions (EAPFs) demonstrated promising anti-SARS-CoV-2 activity at a final concentration of 5.6 μg/mL with a nearly 100% inhibitory effect for *A. vulgaris* (100%), *V. odorata* (100%), and *G. robertianum* (98%) (*p* < 0.05) (Figure 2B). At the same time, the three organic pre-fractions showed reliable cell viability at the two lowest concentration points (*p* < 0.05). The EAPF of *V. odorata* exhibited the most potent antiviral activity, where 1.9 μg/mL reduced the virus-mediated cytopathic effect by more than 85%. In contrast, at concentrations of 50 μg/mL and 100 μg/mL, the cytopathic effect prevailed in all EAPFs so that no antiviral activity could be observed (Figure 2B).

### 3.3 UHPLC–MS chemoprofiles of active crude extracts

**FIGURE 3:**
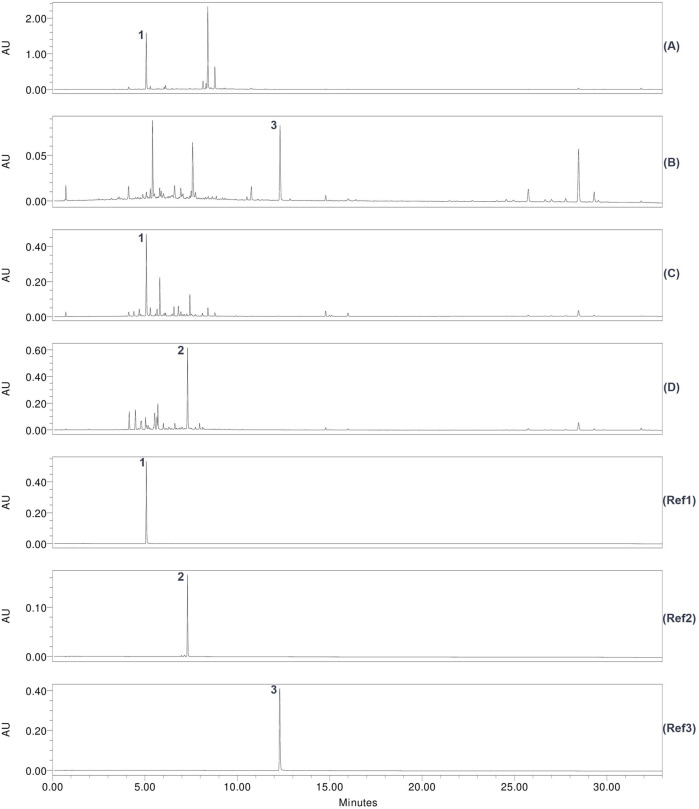
Stacked plots of UHPLC chromatograms at λ = 325 nm. The four top chromatograms show the retained hydroethanolic crude extracts. The three chromatograms below show the reference markers. Reference peaks are numbered based on their elution order. Marker compounds of a reasonable height that were detected in the extract’s chromatograms are numbered according to the reference peak. **(A)** Chromatogram of the *Artemisia vulgaris* hydroethanolic crude extract; **(B)** chromatogram of the *Geranium robertianum* hydroethanolic crude extract; **(C)** chromatogram of the *Sambucus nigra* hydroethanolic crude extract; **(D)** chromatogram of the *Viola odorata* hydroethanolic crude extract. Reference peaks are numbered based on their elution order: Ref 1—chlorogenic acid A 83, Rt = 5.1 min; Ref 2—violanthin, Rt = 7.3 min; Ref 3—kaempferol, Rt = 12.3 min.

Liquid chromatography in combination with mass spectrometry was applied for chemical fingerprinting and to evaluate the presence of marker compounds in the analyzed extracts. The four active crude extracts were characterized according to the requirements for “Extract type C” (see Section 2.4). Correspondingly, a single chemical fingerprint at two detection parameters with three marker compounds was prepared. Chlorogenic acid, kaempferol, and violanthin were chosen as the marker compounds. The extracts were investigated at five wavelengths: 254 nm, 280 nm, 325 nm, 350 nm, and 410 nm (Figure 3; and Supplementary Figures S1-S3, S5).

In all four hydroethanolic extracts, the peaks of the three marker compounds were most pronounced in the chromatogram at λ = 325 nm (Figure 3) and less so at λ = 254 nm, 280 nm, or 350 nm (Supplementary Figures S1-S3). In extracts of both *Artemisia vulgaris* (ArV) and *Sambucus nigra* (SaN), chlorogenic acid (peak 1, Rt 5.1 min) can be detected (Figure 3, chromatograms (A) and (C)). *Viola odorata* extract (ViO) was characterized by the presence of the flavone-C-glycoside violanthin (peak 2, Rt 7.3 min), which shows a UV spectrum characteristic of flavones with absorption maxima at 272 nm and 336 nm (Figure 3, chromatogram (D)). Due to the complexity of the matrix and possible co-eluting peaks in the chromatogram, identification of violanthin was confirmed by MS data with a molecular ion peak of m/z 577 [M-H]^−^ in negative ionization mode and m/z 579 [M + H]^+^ in positive ionization mode. The same retention times, UV spectra, and MS spectra were obtained with the reference substance of violanthin (Supplementary Figure S4). Kaempferol (peak 3, Rt 12.3 min) was only detected in *Geranium robertianum* extract (GeR) (Figure 3, chromatogram (B)).

In the chromatogram at λ = 410 nm, a region of interest extending from 25 min to 32 min was detected in all four extracts (Supplementary Figure S5). Five peaks of reasonable height were detected and numbered according to the elution order (1–5). Peak 3 (Rt = 28.5 min) was found to be of marked height in all four chromatograms. The UV spectrum of Peak 3 revealed two main absorption maxima at 409 nm (Soret band) and 664 nm (Qy band), characteristic of chlorophyll derivative compounds (Supplementary Figure S6).

## 4 Discussion

### 4.1 Historical recipe texts as a resource for this study

Medicinal knowledge in recipe texts is considered largely empirical and thus subject to validation through experience based on the perceived effectiveness, from an emic point of view, of a given therapy (Touwaide, 2007; Oberhelman, 2020; Zipser et al., 2023). Texts of this type stood in dynamic exchange with orally transmitted, constantly evolving knowledge, and therefore, various medical claims listed in them are independent of those in culturally and historically related canonical works (Lardos, 2025). While this has been shown using examples from the Eastern Mediterranean, the same can also be assumed for the case of the RBH. The RBH uses of *Viola odorata* for “figwertzen” or “blattern,” whose possible interpretations include diseases of viral origin (see Table 2), are not found (nor are any closely related uses) in culturally and historically related German herbals, such as prominent texts by Hieronymus Bock (Dobat and Dressendörfer, 2016: chapter CCVII) or Leonhard Fuchs (Hoppe, 1969; chapter CXVII). Therefore, using these herbals, a search for historical plant uses possibly related to viral diseases would not have highlighted *Viola odorata*, which turned out to be one of the most potent plants in our sample.

Moreover, recipe texts provide specific details about the ingredients and the preparation of a given remedy, the possible substitutes to be used, or how the therapy should be carried out. In our sample of 10 plants (Table 1), preparations of the herbal material range from direct applications of the fresh herb and decoctions in water, vegetable oil, or animal fat, to distillation products such as the so-called “burnt waters.”” The latter requires a more sophisticated manufacturing method, as illustrated by the instructions in the RBH on folios 118a and 112v (Fankhauser, 2012) for preparing “eerenpriss wasser” from *Veronica officinalis*: The thoroughly washed fresh herb is first macerated in white wine for 24 h and then it is distilled using a distilling helmet.

The variety and specificity of information in recipe texts can offer various starting points for research questions not only in ethnopharmacology but also in the history of pharmacy or medicine.

However, the investigation of historical medical texts of any type involves numerous challenges, with particularly difficult and complex matters being the assessment of the botanical identities of the mentioned plants and the interpretation of their medicinal uses (Riddle, 2007; Lardos, 2015).

Therefore, we do not claim that the plant identities or the use interpretations offered in our study are definitive. For our conclusions in this respect, we draw on information from the RBH database published by Stehlin et al. (2024), which provides a transparent and traceable data basis. To access and investigate the RBH, Stehlin et al. (2024) followed the recommendations for historical studies by Heinrich et al. (2018). As described in the consensus statement on ethnopharmacological field studies (ConSEFS), the commonly applied method for identifying plants in historical texts relies on authoritative literature that provides botanical identifications for the plant names in question.

To assess the botanical identities of the plants mentioned in the RBH, Stehlin et al. (2024) compiled the identities previously suggested in the literature from three different categories of references: i) linguistic and botanical glossaries that provide scientific names for vernacular plant names in Swiss–German and other German dialects, especially from the late Middle Ages to the Renaissance (Marzell’s, 1943-1979; Fischer, 2001; Schweizerisches Idiotikon, 2023); ii) pharmaco-botanical reference texts associated with the RBH: Leonhard Fuchs’ New Kreuterbůch from 1543 (Dobat and Dressendörfer, 2016) and Hieronymus Bock’s New Kreutterbuch from 1539 and 1546 (Hoppe, 1969); and iii) encyclopedia of the history of pharmaceutical drugs (Schneider, 1974: volume 5, parts 1–3). Any botanical taxon suggested in the literature was regarded as a potential candidate species (Lardos et al., 2024) if the respective plant name corresponded to the plant name appearing in the RBH. By taking into account cultural–historical and plant geographical aspects, along with the agreement between the different categories of references, each candidate species was then assessed for plausibility of attribution: 1—high, 2—moderate, 3—small chance, and ?—doubtful.

To establish the list of plants to be investigated in the present study (see Section 2.1), we only considered the botanical taxon of a particular RBH plant name with the highest probability of correct identification according to Stehlin et al. (2024). For example, the RBH plant name “spitzen wägrich” leads to *Plantago officinalis* L. as the only possible attribution, whereas the case of “voglj krutt” is more complex. Here, the selected candidate species for investigation is the *Caryophyllaceae Stellaria media* (L.) Vill (see Table 1) with a plausibility level of 1; however, several other attributions exist for this name, although with either minor plausibility (*Lysimachia arvensis* (L.) U.Manns & Anderb. (*Primulaceae*), *Senecio vulgaris* L. (*Asteraceae*), *Veronica arvensis* L. (*Plantaginaceae*)), or questionable status (see Supplementary Table S3). these differing attributions reported in the botanical literature used could be related to regional peculiarities or dynamic historical changes in the association of a vernacular plant name with a certain plant species. However, some of these cases may also represent erroneous identifications, which points to the problem of using predefined botanical identities provided in the literature. Because reliance on such existing opinions can be misleading, alternative methods based on systematic comparative analyses of historical plant descriptions with modern botanical morphological information have recently been proposed (Evergetis and Haroutounian, 2015; Lardos et al., 2024).

Although we were able to link the relevant RBH uses to viral diseases, the resources used for the interpretation of the historical disease terms (Höfler, 1970; Riecke, 2004; Schweizerisches Idiotikon, 2023) leave room for other conclusions (see Table 2). In addition to the mentioned diseases of viral origin, the RBH term “figwertzenn” could also refer to syphilitic warts, which are of bacterial origin and caused by the spirochete *Treponema pallidum* ssp. Pallidum (Kang et al., 2019: Chapter 200), in addition to hemorrhoids or other manifestations on the skin. “Blattern” can refer to any type of skin rash with blisters or pustules, boils, or warts, “wolff” can refer to various ulcers or spreading rashes, and “zitter mall” can refer to a wide range of manifestations on the skin, both of infectious or other origin. The most plausible of the interpretations given for “schwartz blattern” is a late stage of smallpox rash with dark discoloration of the crusted lesions. In contrast, hemorrhagic smallpox appears to be less likely as this type of the disease is almost always quickly fatal (Kang et al., 2019: Chapter 166, Poxvirus infections). Another interpretation of this historical term leads to the black lesions of cutaneous anthrax, a bacterial infection caused by *Bacillus anthracis* (Kang et al., 2019: Chapter 156). Overall, these examples suggest that a broad scope of interpretation should generally be applied when elucidating historical concepts of illness.

### 4.2 Forgotten medicinal plants placed in a new context

All 10 plants selected from the Receptarium of Burkhard von Hallwyl (RBH) and investigated in this study are native to the Swiss flora and have a centuries-long history of medicinal use in Europe, sometimes dating back to antiquity, as in the case of *Plantago officinalis*, *Quercus robur*, and *Sambucus nigra* (Dal Cero et al., 2014). While all of the plants in our sample were reported to have been listed in herbals and pharmacopeias from the late medieval and early modern periods in Europe (Schneider, 1974), the majority no longer have official status in today’s evidence-based phytotherapy, as shown by a cross-check with ESCOP (https://www.escop.com/) or HMPC monographs by the European Medicines Agency (EMA) (https://www.ema.europa.eu/). Formerly established medicinal plants may have fallen into disuse due to a lack of evidence for the major indication or because of safety concerns, but often simply because of other reasons unrelated to science (Uehleke et al., 2012). Therefore, the results of our in vitro investigation show that even medicinal plants that are no longer in use could still be interesting candidates for the development of herbal medicines when placed in a new context.

Our results support the view expressed earlier (Buenz et al., 2004) that historical texts on herbal materia medica could be a promising resource for identifying potential candidates for pharmacological investigations. Notably, 4 of the 10 plants selected (40%) display an interesting in vitro anti-SARS-CoV-2 effect: *Artemisia vulgaris* (aerial parts), *Viola odorata* (leaves), *Geranium robertianum* (aerial parts), and *Sambucus nigra* (leaves) (see Figure 1). All four species are wild plants in Switzerland with a relatively wide distribution at lower and medium altitudes. *A. vulgaris* (*Asteraceae*), or common mugwort, is a slightly aromatic perennial that grows on roadsides and riverbanks and is native to the temperate regions of Eurasia. *G. robertianum* (*Geraniaceae*), or herb Robert, is an annual or perennial herb that often grows as a weed in disturbed areas and is widespread throughout the northern hemisphere. *S. nigra* (*Viburnaceae*), or black elderberry, is a small tree native to Europe and Western Asia, best known for the culinary or medicinal uses of its flowers and fruits. *V. odorata* (*Violaceae*), sweet violet, is a perennial herb found in semi-shaded locations, native to Europe, Western Asia, and Northwestern Africa, whose flowers are traditionally processed into perfumes or syrups (Flora, 2024; POWO, 2025).

Specifically, the hydroethanolic extracts and the ethyl acetate pre-fractions from *Viola odorata* and *Geranium robertianum* exhibited a distinct inhibitory activity (Figure 2B). In both plants, we observed a marked protective effect with the ethyl acetate pre-fractions at a concentration of 5.6 μg/mL. At the lowest tested concentration of 1.9 μg/mL, the ethyl acetate pre-fraction (EAPF) of *V. odorata* (leaves) showed a promising antiviral activity of more than 85% without affecting cell viability.

The tested concentration of the positive control, remdesivir (10 µM), corresponds to 6.03 μg/mL (based on the remdesivir molecular mass of 602.585 g/mol). This concentration was selected based on the calculated concentration of the substance in a person’s body fluids (42 L for a 70 kg b.w.), following intravenous administration of the recommended dosage regimen (day 1, 200 mg; days 2–5, 100 mg). The fact that three of our four pre-fractions, which are composed of numerous compounds, exhibited an almost 100% inhibitory activity at a similar concentration (5.6 μg/mL) (see Section 3.2.2) as the single compound remdesivir warrants the call for a detailed fractionation of the active samples.

The pronounced cytopathic effects observed at the highest concentrations of the ethyl acetate pre-fractions (50 µg/mL and 100 μg/mL) (Figure 2B) are related to the assay model, in which the readout for antiviral effects is directly linked to cell viability. As a consequence, the measured values at the respective dosing points do not allow a dose-response curve to be established. To achieve this, more dosing points would be necessary, especially in the lower concentration range.

Our study is also limited in that the antiviral screening approach does not allow for the determination of the selectivity index (SI) as a measure to distinguish true antiviral effects from non-specific cytotoxicity. Despite the promising antiviral effects of three of the four ethyl acetate pre-fractions at 5.6 μg/mL without affecting cell viability (Figure 2B), the significance of the antiviral effects observed should be considered with some reservation. Subsequent work will aim to address this limitation by subjecting the active pre-fractions to detailed fractionation and conducting in-depth investigations of the resulting fractions with regard to antiviral and cytotoxic effects. Only then will it be possible to make more definitive statements about the potential of these candidates.

Finally, it should also be considered that the study employed a clade 19A SARS-CoV-2 strain, which represents a very early lineage of the virus. While the primary objective of the antiviral screening was to identify extracts with broad activity against SARS-CoV-2, it cannot be excluded that the use of other virus variants could have identified a partially different group of active plant extracts. The full scope of the identified extracts' effects against different SARS-CoV-2 variants or other virus types must still be conclusively determined.

Although the remaining 7 of the 11 plants or plant parts tested did not show anti-SARS-CoV-2 activity (or at least not in the assay used), this does not imply that they might not possess antiviral activities against other viruses. Here, it should be noted that this selection of plants originates from RBH recipes associated with viral diseases that cause pathologic manifestations on the skin (Table 2). In fact, published data suggest that some of the plants in question could possess antiviral activities against skin-related conditions, such as ethanolic extracts of *Salvia officinalis* L. against herpes simplex virus types 1 (HSV-1) and 2 (HSV-2) in a plaque reduction assay (Schnitzler et al., 2008), or compounds from *Plantago lanceolata* L. against the monkeypox virus (MPXV) in an *in silico* molecular docking study (Bajrai et al., 2022).

Another possible explanation for the absence of anti-SARS-CoV-2 activity in the seven plants in question is also linked to the selection of the plants and their uses in the RBH. Their uses in the historical text could be associated with viral infections caused by viruses, such as varicella-zoster virus, variola virus, and human papillomavirus (Table 2), which are all DNA viruses (Kang et al., 2019). Coronaviruses, such as SARS-CoV-2, are RNA viruses, which differ significantly from DNA viruses in terms of replication mechanisms. Assuming that our interpretations of the historical uses are plausible and that the historical recipes could have been effective against viral diseases, it is conceivable that the seven plants in question contain compounds that might be active against DNA viruses or RNA viruses other than SARS-CoV-2 by targeting specific molecules involved in the respective viral infection and replication cycles.

### 4.3 Published data on the anti-SARS-CoV-2 effects of the four active plants

Published studies exploring the anti-SARS-CoV-2 effects of the four active plants (*Artemisia vulgaris*, *Geranium robertianum*, *Sambucus nigra*, and *Viola odorata*), if available at all, frequently differed from our study in terms of the plant parts investigated. Therefore, the reported data often can not be directly compared with our results, because flowers, fruits, and leaves differ in their chemical composition. Additionally, some of the studies reported effective concentrations of the samples tested that can be regarded as comparatively high (cf. Gertsch, 2009), and this also may limit comparability.

In traditional Persian medicine, common violet flowers are recommended in pulmonary diseases for the treatment of cough, pneumonia, and pleurisy (Qasemzadeh et al., 2015). In a randomized, double-blind, controlled trial assessing the effects of *Viola odorata* syrup in conjunction with the conventional protocol, faster recovery and alleviation of respiratory symptoms were observed in patients who tested for SARS-CoV-2 (Mehraban et al., 2023). However, no clinical studies have been conducted on the anti-SARS-CoV-2 effects of *Viola odorata* leaves investigated in our study. Furthermore, specific antiviral data for extracts of different parts of *V. odorata* appear to be lacking in general. Only biological activity data regarding the antioxidant potential of the leaves of this species are available (Akhbari et al., 2012; Habibi et al., 2019; Aslam et al., 2020).

To date, no data are available on the potential of *Geranium robertianum* against SARS-CoV-2. However, because our hydroethanolic extract of *G. robertianum*, in particular, is estimated to be rich in polyphenols, as suggested by the UV spectra of the majority of the detected peaks (data not shown), the following study, although conducted with an East Asian *Geranium* species, is worth mentioning. The ethanolic extract of *Geranii Herba*, specified as the aerial parts of *Geranium thunbergii* Siebold & Zucc (tested at 100 µg/mL and 200 μg/mL) and nine polyphenolic compounds identified in the extract (gallic acid, protocatechuic acid, corilagin, geraniin, ellagic acid, kaempferitrin, kaempferol 7-O-rhamnoside, quercetin, and kaempferol, all tested at 100 μM), were reported to exhibit antiviral activity against strains of Influenza A in MDCK cells and human lung epithelial cells, most potently through the inhibition of neuraminidase. Of the compounds tested, geraniin showed the highest neuraminidase inhibitory activity (Choi et al., 2019). Using the same nine polyphenolic compounds from *Geranii Herba*, Arokiyaraj et al. (2020) conducted an *in silico* study to investigate the compounds’ potential activity against SARS-CoV-2. The results of the docking study indicated that the compounds interacted with viral target proteins, especially the compound geraniin, which showed a significant binding affinity to the spike protein. A review of the European species *G. sanguineum* L. regarding ubiquitous polyphenols with reported anti-SARS-CoV-2 activity (Abarova et al., 2024) further supports the assumption that this class of compounds could play a role in the antiviral activity of *Geranium* species in general.

Data on potential anti-SARS-CoV-2 effects are available for the flowers and fruits of *Sambucus nigra*, but they were not available for the leaves of this species, the plant part investigated in this study. Leaf extracts were investigated, for example, for their *in vitro* anti-inflammatory and antioxidant properties (Skowrońska et al., 2022) or their wound-healing potential (Skowrońska et al., 2024). A combination of *S. nigra* fruit extract and quinine was reported to exhibit strong antiviral effects against both Influenza A virus (in MDCK cells) and SARS-CoV-2 (in human lung epithelial cells) (Setz et al., 2025). The anti-SARS-CoV-2 effects of different common medicinal plants, including *S. nigra* flowers, were also assessed in a Vero E6 cell model, in which a protection against the virus-induced cytopathic effect was reported at extract concentrations of >50 μg/mL (Leka et al., 2022). In another Vero E6 cell assay, the juice of *S. nigra* fruits was found to exhibit strong virucidal activity against the influenza A virus only, with no effect against adenovirus Type 5 or SARS-CoV-2 (Eggers et al., 2022). Conversely, for both *S. nigra* flower and fruit extracts, inhibitory capacities toward SARS-CoV-2 surface protein binding and receptor binding domains were observed in a cell-free assay (Boroduske et al., 2021). Although no other study has investigated the anti-SARS-CoV-2 potential of *S. nigra* leaves to date, the presence of cyanogenic glycosides such as sambunigrin (Młynarczyk et al., 2018) should be taken into account due to its known toxicity in any further development of the extract or its pre-fractions.

*Artemisia vulgaris* was found to be the most potent species of six different *Artemisia* species in terms of a direct inactivation (neutralization) of SARS-CoV-2 in a Vero E6 cell model, exhibiting IC_50_ values ranging from 1.1 μg/mL to 14.65 μg/mL for the ethanolic extract of the plant parts tested (flowers, leaves, and stems) (Kazachinskaia et al., 2022). The results of another Vero E6 cell assay pointed to the potential importance of endophytic fungi in the leaves and stems of *A. vulgaris* in the plant’s anti-SARS-CoV-2 effect (Maehara et al., 2023). Results from our preliminary studies on respiratory epithelial cell assays also suggest that the antiviral effect observed in our study is based on a virus-neutralization mechanism, as the extracts and pre-fractions investigated were only effective when pre-incubated with the virus, and not when pre-incubated with the cells (unpublished data). The possibility of an antiviral activity based on a virus-neutralization effect prior to entry into the host cell could represent a starting point for therapeutic strategies.

### 4.4 Current knowledge on the phytochemical composition of the four active plants

Based on the extraction solvent (80% ethanol) applied to the four active extracts, it can be assumed that polar and semi-polar constituents from *Artemisia vulgaris*, *Geranium robertianum*, *Sambucus nigra*, and *Viola odorata* can be recovered. These include, for example, ubiquitous flavonoids such as quercetin glycosides, kaempferol derivatives, luteolin, apigenin, or phenolic acids such as caffeic acid, chlorogenic acid, or dicaffeoylquinic acids (Alara et al., 2021; Jovanović et al., 2015).

The aerial parts of *Artemisia vulgaris* contain a mixture of secondary metabolites, with several major compound classes, including, for example, sesquiterpene lactones, flavonoids, coumarins, phenolic acids, and mono- and sesquiterpenes (Abiri et al., 2018; Ekiert et al., 2020). Studies have reported on specific sesquiterpene lactones, such as vulgarin, psilostachyin, and psilostachyin C (Geissman and Ellestad, 1962; Marco et al., 1991), along with small but detectable quantities of artemisinin (Saraji et al., 2013). Artemisinin, originally isolated from *Artemisia annua* L., has been investigated for its anti-SARS-CoV-2 potential in different cell lines (Zhou et al., 2021). Flavonoids, such as kaempferol and quercetin derivatives, or phenolic acids, such as caffeic acid, chlorogenic acid, and dicaffeoylquinic acid isomers, were also detected (Lee et al., 1998; Ivanescu et al., 2010). Among the coumarins identified in the aerial parts of *Artemisia vulgaris*, umbelliferone, a moderately polar constituent, may be extractable in an 80% ethanolic macerate and has been documented (*in silico*) to have anti-SARS-CoV-2 activity (Cheng et al., 2023).

In *G. robertianum*, mainly flavonoids, tannins, and phenolic acids have been described (Graça et al., 2016). The hydrolyzable tannin geraniin is generally found in the genus *Geranium* (Harborne and Williams, 2002). Several *in vitro* studies have reported the antiviral properties of geraniin against upper respiratory viruses (Ramesh and Valan, 2021; Wang et al., 2024), and the compound has been found to block the interaction between the viral spike protein and the human ACE2 receptor, thereby inhibiting viral entry into host cells (Kim et al., 2021). Further important phenolic compounds in *G. robertianum* are gallic and ellagic acids (Graça et al., 2016), with gallic acid being reported in different polar extracts of the species (Neagu et al., 2017). For ellagic acid, anti-SARS-CoV-2 effects have been reported in several *in vitro* studies, but its potential and mechanism require further clarification (Sayed et al., 2020; Umar et al., 2022; Alexova et al., 2023; Fatiningtya et al., 2024; Wichian et al., 2025). Unlike the flowers and fruits, the chemical composition of the leaves of *Sambucus nigra*, as used in our study, has been less extensively studied. Mainly phenolic compounds were detected in the leaves to date, including flavonoids such as quercetin, rutin, isoquercitrin, kaempferol, and astragalin, along with caffeic acid derivatives, dicaffeoylquinic acid isomers, neochlorogenic acid, or other phenolic derivatives (Tundis et al., 2019; Skowrońska et al., 2022; Sánchez-Hernández et al., 2023). Toxic cyanogenic glycosides also identified in leaves of *S. nigra*, as, for example, sambunigrin (Młynarczyk et al., 2018), are expected to be retained in ethanolic extracts prepared at room temperature (Skowrońska et al., 2022), such as the leaf extract investigated in our study, and therefore deserve special attention (see Section 4.3).

In a review of the chemical composition of *Viola odorata*, 83 compounds were highlighted, many of which belong to the cyclotides, a class of proteins broadly represented in *Violaceae* and identified in the aerial parts of the species (Fernández-zey et al., 2024). *Violaceae* are also known to possess numerous cyclotides, such as cycloviolacin VY1. The compound, isolated from the entire plant of *Viola yedoensis* Makino Bot. Mag. (accepted name *Viola philippica* var. philippica), has been reported to exhibit activity against the influenza A H1N1 virus (Liu et al., 2014; Mammari et al., 2021). Cycloviolacin peptides have also been investigated *in silico* for their anti-SARS-CoV-2 potential (Bhandu et al., 2023). Various compounds reported in *V. odorata* were also identified, specifically in the leaves, which are the plant parts used in the respective extract of our study. This includes phenolic compounds and flavonoids such as catechin (Jurca, 2019), but also, for example, quercetin, caffeic acid, chlorogenic acid, and ferulic acid (Jamshed et al., 2019; Sikandar et al., 2021). Phenolic compounds have also been reported in the leaves of seven *Viola* species from China (Zeng et al., 2025) and appear to be substantially recovered in intermediate-polarity extracts, such as methanolic or ethanolic extracts and their fractions (Aslam et al., 2020; Sikandar et al., 2021). Therefore, it can be assumed that phenolic compounds are also a relevant compound class in the ethanolic leaf extract of *V. odorata* investigated in our study.

Of the 67 different flavonoids identified in the *Viola* genus (Zhang et al., 2023), the flavone-glycoside violanthin has been identified in the entire plant of *V. tricolor* L., the flowers of *V. etrusca* Erben, and the aerial parts of *V. arvensis* Murray (Fernández-Bobey et al., 2024), but until our study, apparently not in *Viola odorata* (see Section 4.5). Further flavonoids, such as isovitexin, kaempferol-6-glucoside, and rutin, have been reported in an ethanolic extract of *V. odorata* aerial parts (Erdogan Orhan et al., 2015), which presumably contained a relevant proportion of leaves. In the leaves of the species, tannins have also been detected, with a total tannin content of 42.35 mg/g expressed as gallic acid equivalents (Aslam et al., 2020; Dastagir et al., 2023), along with the widely distributed phytosterol stigmasterol (Aslam et al., 2020). Various in silico studies have predicted that phytosterols may interfere with several SARS-CoV-2 target proteins (Bondhon et al., 2021; Obakan Yerlikaya et al., 2023; Wahyuni et al., 2023).

### 4.5 Phytochemical fingerprints of the four active extracts

To ensure the reproducibility of results in medicinal plant research, it is essential to provide a phytochemical characterization of the plant extracts under investigation. According to best practice recommendations for the chemical characterization of herbal extracts by Heinrich et al. (2022), different requirements exist depending on a plant’s regulatory status and importance in international trade. For extracts of plants that are neither included in pharmacopeias nor commercially used at an international level (classified as “type C extracts”), a single chemical fingerprint with one or more detection parameters and, if possible, a description of marker compounds is recommended. We followed the recommendations for extracts of type C because the herbal substances of the four active extracts–the leaves of *S. nigra* and *V. odorata,* and the aerial parts of *A. vulgaris* and *G. robertianum*—i) are not listed in current national or regional pharmacopeias as starting materials for herbal medicinal products and ii) are not subject to international trade.

Three different marker compounds were associated with one or more of the four active crude extracts characterized by a phytochemical fingerprint, including chlorogenic acid, kaempferol, and violanthin (Section 3.4).

Chlorogenic acid, a widely distributed phenolic acid, was detected in the hydroethanolic extracts of *Artemisia vulgaris* (aerial parts) and *Sambucus nigra* (leaves) (Figure 3). Its presence in the two species and their respective plant parts has also been reported in other studies (Tundis et al., 2019; Melguizo-Melguizo et al., 2014). Chlorogenic acid has been reported to exhibit anti-SARS-CoV-2 activity *in vitro* (Bahun et al., 2022; Wang et al., 2022; Shafiq et al., 2023) and activity against influenza in both cellular and animal models (Karar et al., 2016; Aljehany, 2022).

Kaempferol, the marker compound detected in the hydroethanolic extract of *Geranium robertianum*, constitutes, together with quercetin and the glycosides, the main flavonoid found in the species (Graça et al., 2016). Kaempferol and its glycosides have been investigated for their inhibitory effects against SARS-CoV-2 and the influenza virus (Periferakis et al., 2023; Liu et al., 2008; Anand et al., 2021). The compound has also been the subject of mechanistic studies on SARS-CoV-2 (Sopjani et al., 2024) and may contribute to COVID-19 prevention and treatment by targeting pathways involved in endotoxin-induced cytokine storms, thereby acting as an anti-inflammatory factor (Sun et al., 2023; Khalil et al., 2020).

Violanthin, the flavone-glycoside used as a marker compound in the hydroethanolic extract of *V. odorata*, is, to the best of our knowledge, reported here for the first time in the leaves of this species. Being a constituent of a polyherbal formulation from India, violanthin was assessed *in silico* for its anti-SARS-CoV-2 potential and suggested to exhibit binding affinity for SARS-CoV-2 virus 3CL-like proteases (Vincent et al., 2020). Another *in silico* study of Unani medicinal plants found that emetine and triacetonamine, two compounds also found in *Viola odorata*, interfere with SARS-CoV-2. While triacetonamine showed only limited binding affinity with viral components, the alkaloid emetine effectively appeared to inhibit the binding of the spike protein to host cells (Anwar et al., 2024). Previous studies have suggested different mechanisms of anti-SARS-CoV-2 activities for emetine, including its binding affinity to the virus’s main protease (Mpro) and papain-like protease (PLpro) (Zhang et al., 2022).

Taken together, the previously published data suggest a possible contribution of the three highlighted marker compounds to the anti-SARS-CoV-2 activities observed with the four active crude extracts (*A. vulgaris*, *G. robertianum*, *S. nigra*, and *V. odorata*).

## 5 Conclusion

This study builds on previous examples that illustrate the potential of using premodern written sources as a starting point for pharmacological investigations. Using a systematic approach to identify relevant medicinal plant uses in the historical text, we selected 10 plant species to test against SARS-CoV-2, of which 4—*Artemisia vulgaris*, *Geranium robertianum*, *Sambucus nigra* (leaves), and *Viola odorata* (leaves)—showed promising bioactivity profiles.

Although vaccination is, to date, the most important preventive measure against SARS-CoV-2, the emergence of new circulating variants poses a constant threat to the effectiveness of available vaccines. Complementary preventive measures, ideally at the local entrance points of the pathogen, could help to further reduce the risk of infection. In this context, natural antiviral agents that act as viral entrance inhibitors might constitute a promising avenue. From a methodological point of view, the study shows the challenges associated with the investigation of historical texts. These particularly concern the interpretation of historical disease terms and their translation into modern clinical terminology, along with the assessment of the botanical identities of the historical plant names. We bin no way claim to have the final word regarding the interpretation of the historical uses or the botanical identification of the plant names in the RBH. Furthermore, our plant selection is not based on a direct correlation with historical plant uses based on the organ system treated, which in the case of COVID-19 is the respiratory system, but rather on the pharmacological property in question, in this case: a putative antiviral activity. Nevertheless, the comparatively high proportion of active plants in our sample underpins the validity of our method of selecting possible candidates from the historical text.

The significance of the antiviral effects observed in our study should be considered with some reservations due to methodological limitations. In addition, the preliminary characterization of the extract-based chromatographic fingerprints makes it difficult to draw more precise conclusions about the involvement of specific plant compounds in the observed activities. While the principal aim of this study was the ethnopharmacological identification of plants described in the RBH with a possible connection to viral diseases and their investigation in a first antiviral screening, it will be the aim of subsequent work to address the existing gaps. Only then will it be possible to make more definitive statements about the potential of these “forgotten” medicinal plants.

## Data Availability

The original contributions presented in the study are included in the article/Supplementary Material; further inquiries can be directed to the corresponding author.
