# Supplementary material for: Historical texts as a potential resource for plant-based antiviral agents against SARS-CoV-2: the example of the Receptarium of Burkhard III von Hallwyl from 16th-century Switzerland
Source: Front Pharmacol. 2026 Feb 19;16:1731629. doi: 10.3389/fphar.2025.1731629 (PMC12960544; doi:10.3389/fphar.2025.1731629)
Supplement: Supplementary file 1 [file DataSheet1.pdf]

Supplementary Material

Historical texts as a potential resource for plant-based antiviral agents against SARS-CoV-2 – the example of the *Receptarium* of Burkhard III von Hallwyl from 16th century Switzerland

Nina Vahekeni[1], Jonas Stehlin[1], Corinna Urmann[2,3], Evelyn Wolfram[1], Yannick Geissmann[4], Yelena Ruedin[4], Samuel Peter[1], Olivier Engler[4], Andreas Lardos[1*]

***Correspondence:** Andreas Lardos, andreas.lardos@zhaw.ch

Table S1. Specification of the 11 bulk samples of plant material

Plant species	Plant part	Plant material ID	Supplier	Supplier lot no.
Alchemilla vulgaris L.	aerial part	20220008	Dixa AG	211241
Artemisia vulgaris L.[1]	aerial part	20220015	Dixa AG	214542
Geranium robertianum L.[1]	aerial part	20220010	Dixa AG	212663
Plantago lanceolata L.	leaves	20220001	Dixa AG	192663
Quercus robur L.	leaves	20220004	Dixa AG	202156
Salvia officinalis L.[1]	leaves	20220007	Dixa AG	210630
Sambucus nigra L.	leaves	20220005	Dixa AG	203084
Stellaria media (L.) Vill.[1]	aerial part	20220009	Dixa AG	211824
Veronica officinalis L.[1]	aerial part	20220011	Dixa AG	213169
Viola odorata L.	flowers	20220012	Dixa AG	213691
Viola odorata L.	leaves	20220003	Dixa AG	201667

[1]In cases where the plant part to be used according to the respective RBH recipe (see Table 1) was either not available (as in the case of the “tip of shoots” of *Artemisia vulgaris*) or not specified, the plant part commonly used in herbal medicine was obtained. For *Viola odorata*, two different plant parts were obtained (flowers and leaves), because the part specified in RBH ("aerial part", see Table 1) was not available.

Table S2. Chemicals and consumables

Article name	Supplier	Article no.	Supplier lot no.
Ethanol absolute	VWR	20821.330	23B224007
Ethyl acetate	VWR	23882.321	22G294044
Filter paper	Macherey-Nagel	729245.400	0.337

Table S3. Selecting the plant use record from the RBH database (Stehlin et al., 2024) for further investigation. Only plant use records are listed which refer to historical uses whose interpretations include diseases of viral origin according to Stehlin et al. (2024).

Selected use record	RBH plant name	Part used	Use in RBH	Preparation in RBH	Recipe signature	Candidate species
Yes	sinauw	Not mentioned	figwertzenn	Decoction in animal fat	120b,115r.02	*Alchemilla xanthochlora* agg. (incl. *A. vulgaris* L., *A. xanthochlora* Rothm.) (1)
Yes	vnnserr frouwen mentle	Not mentioned	figwertzenn	Distillate ('burnt water')	122a,116v.02	*Alchemilla xanthochlora* agg. (incl. *A. vulgaris* L., *A. xanthochlora* Rothm.) (1)
Yes	gotz gnad	Not mentioned	figwertzenn	Heat bag with the fresh herb	121b,116r.05	*Geranium robertianum* L. (1); *Geranium pratense* L., *Gratiola officinalis* L. (2); *Geranium* spp. ?
Yes	gotz gnad	Not mentioned	figwertzenn	Distillate ('burnt water')	121b,116r.06	*Geranium robertianum* L. (1); *Geranium pratense* L., *Gratiola officinalis* L. (2); *Geranium* spp. ?
Yes	rot bugglenn	Tip of shoot	figwertzenn	Decoction in olive oil	120b,115r.03.v01	*Artemisia vulgaris* L. (1); *Artemisia campestris* L.?, *Amaranthus blitum* L.?, *Rumex obtusifolius* L.?, *Portulaca oleracea* L.?
Yes	spitzen wägrich	Not mentioned	wolff	Juice of fresh herb mixed with honey and butter	114b,109r.05.v02	*Plantago lanceolata* L. (1)
Yes	eichen	Leaves	figwertzenn	Cataplasm of the fresh herb	120b,115r.05.v03	*Quercus robur* L., *Q. petraea* (Matt.) Liebl. (1), *Quercus* spp.?
Yes	edle salbinen	Not mentioned	figwertzenn	Decoction in olive oil	120b,115r.03.v01	*Salvia officinalis* L. (1)
Yes	holder	Leaves	blattern	Juice of the fresh herb	107a,101v.08.v02	*Sambucus nigra* L. (1)
Yes	voglj krutt	Aerial part	figwertzenn	Decoction in animal fat	120b,115r.02	*Stellaria media* (L.) Vill. (1), *Lysimachia arvensis* (L.) U.Manns & Anderb. (*Anagallis arvensis* L.) (2); *Senecio vulgaris* L., *Veronica arvensis* L. (3), *Geranium robertianum* L.?, *Polygonum aviculare* L.?, *Legousia speculum-veneris* (L.) Durande?, other less likely taxa
Yes	eerenpriss	Not mentioned	zitter mall	Distillate ('burnt water') mixed with vitriol	134a,128v.08.v01	*Veronica officinalis* L. (1); *V. chamaedrys* L.?, *V. hederifolia* L.?, *V. arvensis* L.?, *V. serpyllifolia* L.?
Yes	blauw viönlin	Not mentioned	figwertzenn	Macerate in olive oil	120b,115r.01	*Viola odorata* L. (1); *Hesperis matronalis* L.?
Yes	blauw viönlin	Not mentioned	figwertzenn	Decoction in water	122a,116v.01	*Viola odorata* L. (1); *Hesperis matronalis* L.?
Yes	viöl	Aerial part	figwertzenn	Decoction in water	121b,116r.03	*Viola odorata* L. (1); *Viola tricolor* L. (2); *Viola* spp.?

frontiers

Selected use record	RBH plant name	Part used	Use in RBH	Preparation in RBH	Recipe signature	Candidate species
Yes	viönlin	Not mentioned	figwertzenn	Macerate in olive oil	121a,115v.02	<u>*Viola odorata* L.</u> (1); *Iris germanica* L., *I. florentina* L.; *Matthiola incana* L., *Erysimum cheiri* (L.) Crantz (*Cheiranthus cheiri* L.) (2); *Hesperis matronalis* L., *Matthiola annua* Sw. (3)
Yes	vigell	Not mentioned	blattern	Macerate in olive oil	107a,101v.08.v02	<u>*Viola odorata* L.</u> (1); *Iris germanica* L., *I. florentina* L.; *Matthiola incana* L., *Erysimum cheiri* (L.) Crantz (*Cheiranthus cheiri* L.) (2); *Hesperis matronalis* L., *Matthiola annua* Sw. (3)
No, plant not available	weid eschenn	Not mentioned	figwertzenn	Decoction in water together with quicklime	121b,116r.01	<u>*Fraxinus excelsior* L.</u> (1)
No, plant not available	wÿss gilgen	Leaves	schwartz blatteren	Juice of fresh herb	136a,130v.02.v02	<u>*Lilium candidum* L.</u> (1)
No, plant not available	fünff finngerr krutt	Not mentioned	figwertzenn	Hot cataplasm of the powdered herb	121a,115v.05	<u>*Potentilla reptans* L.</u> (1); *Potentilla recta* L., *P. erecta* (L.) Raeusch (2); *Potentilla* spp. (3)
No, plant not available	fünff finngerr krutt	Root	figwertzenn	Cataplasm of the powdered herb	121a,115v.06	<u>*Potentilla reptans* L.</u> (1); *Potentilla recta* L., *P. erecta* (L.) Raeusch (2); *Potentilla* spp. (3)
No, plant not available	ruten	Seed	zitter mall	Chewed and mixed with saliva	134a,128v.03	<u>*Ruta graveolens* L.</u> (1)
No, plant not available	bach bumlen	Not mentioned	figwertzenn	Hot cataplasm with the juice of the fresh herb mixed with butter	121b,116r.03.z01	<u>*Veronica beccabunga* L.</u> (1); *Veronica anagallis-aquatica* L. (2); *Caltha palustris* L.?, *Trollius europaeus* L.?
No, plant identity uncertain	müllj stoub	Seed (flour)	figwertzenn	Cataplasm of the powdered herb mixed with olive oil	121a,115v.01	Flour dust (e.g. from *Triticum* spp.) (2)
No, plant identity uncertain	glariett	Gum, resin	Blattern	Plant exudate is washed in water and mixed with butter	105a,99v.02	*Larix decidua* Mill., *Prunus avium* L. (3); *Prunus* spp.?
No, plant identity uncertain	für blumen	Not mentioned	figwertzenn	Macerate in olive oil	120b,115r.01	*Papaver rhoeas* L.?, *P. dubium* L.?, *P. argemone* L.?, *Silene dioica* (L.) Clairv.?, *Silene flos-cuculi* (L.) Clairv.?, *Adonis aestivalis* L.?, *Lilium bulbiferum* L.?, *Primula farinosa* L.?
No, plant identity uncertain	ross	Not mentioned	Blattern	Macerate in olive oil	107a,101v.08.v02	*Rosa gallica* L, *R.* x *alba* L., *R.* x *damascena* Herrm., *R.* x *centifolia* L. (1); *Rosa* spp.?
No, plant identity uncertain, plant not available	lilienn	Flower	zitter mall	Flower water	134a,128v.05	*Lilium candidum* L. (3); *Iris germanica* L.?, *Iris* spp.?

Selected use record	RBH plant name	Part used	Use in RBH	Preparation in RBH	Recipe signature	Candidate species
No, plant identity uncertain, plant not available	holtz öpffell	Not mentioned	figwertzenn	Juice of the fresh herb as ingredient in compound ointment	121a,115v.03	*Malus sylvestris* (L.) Mill. (2); *Picea abies* (L.) H.Karst.?
No, plant identity uncertain, plant not available	knaben krutt	Bulb	figwertzenn	Decoction in wine	121a,115v.04	*Orchis mascula* L., *O. militaris* L., *O. morio* L., *O. ustulata* L., *Dactylorhiza maculata* (L.) Soo agg., *Gymnadenia conopsea* (L.) R.Br., *Platanthera bifolia* (L.). Rich. (1); *Hylotelephium telephium* (L.) H.Ohba (*Sedum telephium* L.) (2); further orchids?
No, plant identity uncertain, plant not available	sigilis solomonis	Root	zitter mall	Decoction of the fresh herb in honey	134a,128v.06	*Polygonatum odoratum* (Mill.) Druce, *P. multiflorum* (L.) All., *P. verticillatum* (L.) AlL. (1)
No, pant identity uncertain, plant not available	nachtschatten	Root	figwertzenn	Juice of the fresh herb as ingredient in compound ointment	121b,116r.02	*Solanum nigrum* L., *S. dulcamara* L. (1)

Selected use record: Only use records referring to RBH plant names which can be mapped to one specific botanical taxon of high plausibility of being the correct identification (see column "Candidate species") were selected for further investigation. Use records associated with candidate species of minor plausibility were excluded (indicated as not selected). Furthermore, use records referring to candidate species that were not commercially available were also excluded (indicated as not selected).

RBH plant name: Plant name as stated in the respective use record of the RBH database (Stehlin et al., 2024: Table 2).

RBH plant part: Plant part as stated in the respective use record of the RBH database (Stehlin et al., 2024: Table 2).

RBH use: Historical uses as stated in the respective use record of the RBH database (Stehlin et al., 2024: Table 1).

Preparation in RBH: Preparation of the herb described in the recipe associated with the respective use record in the RBH database (Stehlin et al., 2024).

Recipe signature: Identifier of the respective recipe in the RBH database (Stehlin et al., 2024).

Candidate species: Scientific names of candidate plants for the RBH plant name, adopted from Stehlin et al. (2024: Table 2). The rating of the candidate plants regarding the plausibility of being the correct attribution (1 – high, 2 – moderate, 3 – small chance, ? – doubtful) were likewise adopted from Stehlin et al. (2024, see section 2.2.2). RBH plant names which can be mapped to one specific botanical taxon of high plausibility of being the correct identification (plausibility level 1) are underlined.

Table S4. Specification of the 22 samples of crude extracts prepared from the 11 bulk samples of plant material specified in **Table S1**.

**Plant species**	**Plant part**	**Plant material ID**	**Extraction solvent**	**Extraction yield (%)[1]**
Alchemilla vulgaris L.	aerial part	20220008	H_2O	22
Artemisia vulgaris L.	aerial part	20220015	H_2O	21
Geranium robertianum L.	aerial part	20220010	H_2O	24
Plantago lanceolata L.	leaves	20220001	H_2O	29
Quercus robur L.	leaves	20220004	H_2O	23
Salvia officinalis L.	leaves	20220007	H_2O	16
Sambucus nigra L.	leaves	20220005	H_2O	26
Stellaria media (L.) Vill.	aerial part	20220009	H_2O	25
Veronica officinalis L.	aerial part	20220011	H_2O	25
Viola odorata L.	flowers	20220012	H_2O	31
Viola odorata L.	leaves	20220003	H_2O	19
Alchemilla vulgaris L.	aerial part	20220008	EtOH 80%	12
Artemisia vulgaris L.	aerial part	20220015	EtOH 80%	14
Geranium robertianum L.	aerial part	20220010	EtOH 80%	12
Plantago lanceolata L.	leaves	20220001	EtOH 80%	33
Quercus robur L.	leaves	20220004	EtOH 80%	22
Salvia officinalis L.	leaves	20220007	EtOH 80%	16
Sambucus nigra L.	leaves	20220005	EtOH 80%	20
Stellaria media (L.) Vill.	aerial part	20220009	EtOH 80%	14
Veronica officinalis L.	aerial part	20220011	EtOH 80%	20
Viola odorata L.	flowers	20220012	EtOH 80%	28
Viola odorata L.	leaves	20220003	EtOH 80%	12

[1]Extraction yield (%) = [Dried extract weight / Dried plant material weight * 100]

Table S5. Antiviral screening of the 22 samples of crude extracts prepared from the 11 bulk samples of plant material specified in **Table S1**.

**Plant species**[a]	**Plant part**[a]	**Extraction solvent**[a]	**Antiviral activity at 16,7 µg/mL [%]**[b]	**Cell viability at 16,7 µg/mL [%]**[c]
Alchemilla vulgaris L.	aerial part	H_2O	-0,64%	89,76%
Artemisia vulgaris L.	aerial part	H_2O	-0,95%	99,92%
Geranium robertianum L.	aerial part	H_2O	-4,38%	89,57%
Plantago lanceolata L.	leaves	H_2O	1.08%	101.41%
Quercus robur L.	leaves	H_2O	0,92%	90,97%
Salvia officinalis L.	leaves	H_2O	-14,30%	92,84%
Sambucus nigra L.	leaves	H_2O	-2,58%	98,53%
Stellaria media (L.) Vill.	aerial part	H_2O	-1,45%	95,96%
Veronica officinalis L.	aerial part	H_2O	-3,26%	99,48%
Viola odorata L.	flowers	H_2O	-6,57%	100,10%
Viola odorata L.	leaves	H_2O	0,41%	98,56%
Alchemilla vulgaris L.	aerial part	EtOH 80%	29,78%	**61.94%**
Artemisia vulgaris L.	aerial part	EtOH 80%	**100.12%**	105.36%
Geranium robertianum L.	aerial part	EtOH 80%	**90.12%**	100.68%
Plantago lanceolata L.	leaves	EtOH 80%	-0.62%	127.98%
Quercus robur L.	leaves	EtOH 80%	-5,62%	89.15%
Salvia officinalis L.	leaves	EtOH 80%	-5,43%	92.90%
Sambucus nigra L.	leaves	EtOH 80%	**93.71%**	98.34%
Stellaria media (L.) Vill.	aerial part	EtOH 80%	32,14%	107,02%
Veronica officinalis L.	aerial part	EtOH 80%	36,14%	97,40%
Viola odorata L.	flowers	EtOH 80%	-2,55%	99,47%
Viola odorata L.	leaves	EtOH 80%	**93.81%**	107,03%

[a] Plant material and solvents used for the preparation of the crude extracts.
[b] The antiviral activity measures the inhibition of the cytopathic effect (CPE). Measurements were normalized to virus control and cell control as 0% and 100% respectively. Samples displaying an antiviral activity of ≥ 50% at 16.7 µg/mL are highlighted in grey.
[c] Cell viability measurements were normalized to cell control as 100%. Values of less than 75% were considered as cytotoxic and corresponding samples excluded from further investigation (see *Alchemilla vulgaris* aqueous extract displaying a cell viability of 61.94% at 16.7 µg/mL).

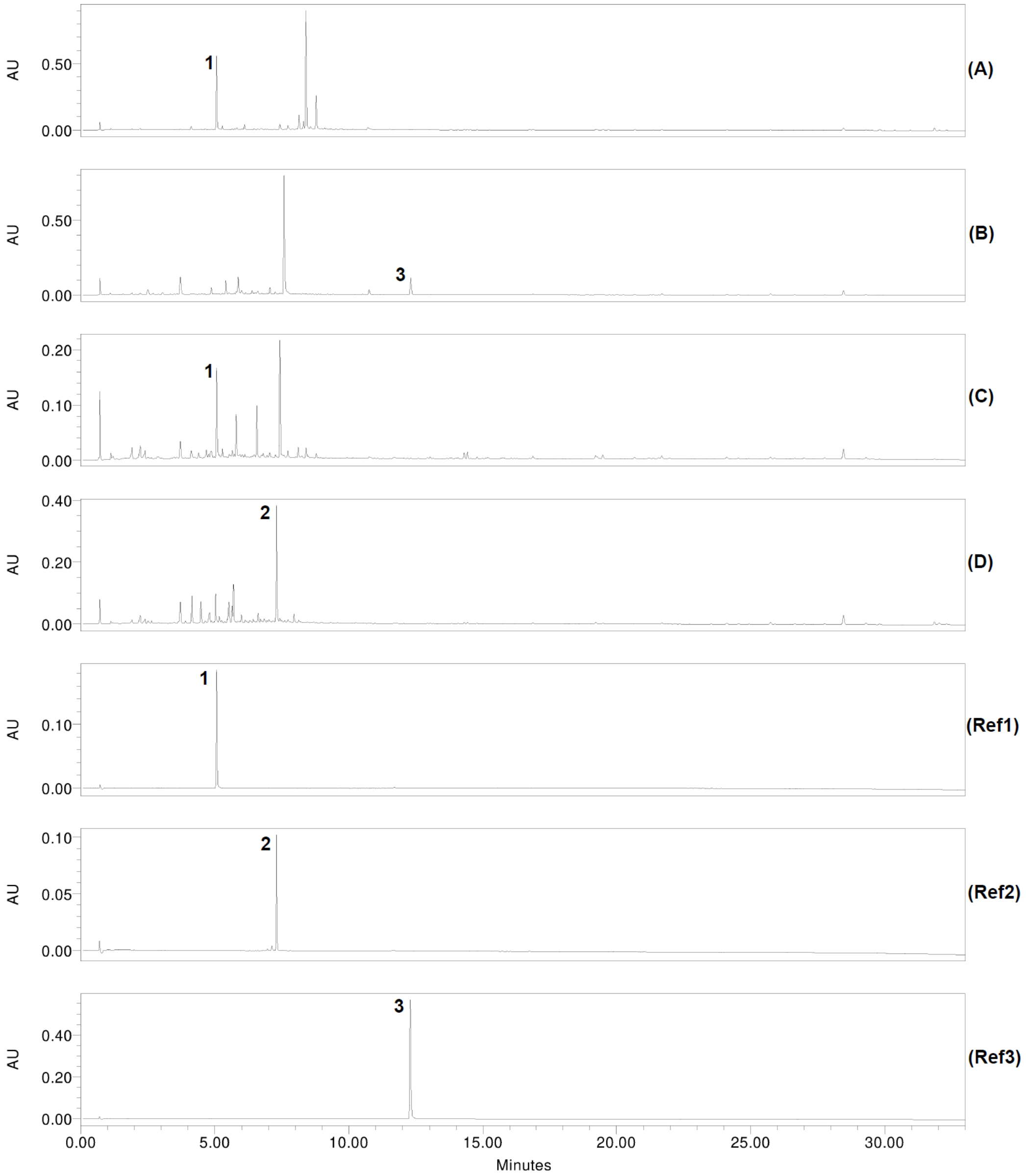

Figure S1. Stacked plots of UHPLC chromatograms at λ=254 nm. The four top chromatograms show the retained hydroethanolic crude extracts. The three chromatograms below show the reference markers. Reference peaks were numbered based on their elution order. Marker compounds of a reasonable height that were detected in the extract's chromatograms were numbered according to the reference peak. (A) chromatogram of *Artemisia vulgaris* hydroethanolic crude extract; (B) chromatogram of *Geranium robertianum* hydroethanolic crude extract; (C) chromatogram of *Sambucus nigra* hydroethanolic crude extract; (D) chromatogram of *Viola odorata* hydroethanolic crude extract. Reference peaks were numbered based on their elution order: Ref 1 – Chlorogenic acid A 83, Rt=5.1 min.; Ref 2 – Violanthin, Rt=7.3 min.; Ref 3 – Kaempferol, Rt=12.3 min.

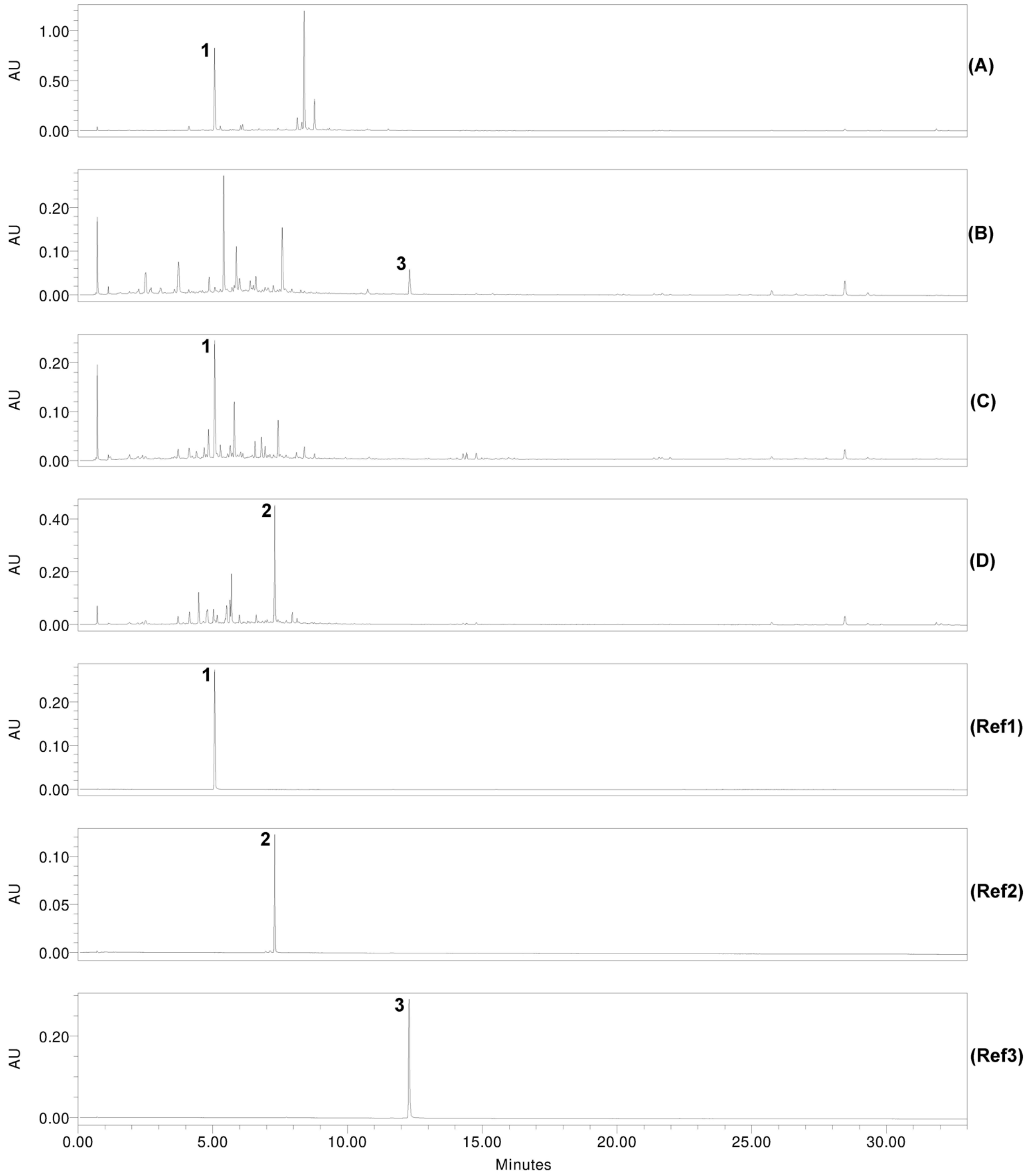

Figure S2. Stacked plots of UHPLC chromatograms at λ=280 nm. The four top chromatograms show the retained hydroethanolic crude extracts. The three chromatograms below show the reference markers. Reference peaks were numbered based on their elution order. Marker compounds of a reasonable height that were detected in the extract's chromatograms were numbered according to the reference peak. (A) chromatogram of *Artemisia vulgaris* hydroethanolic crude extract; (B) chromatogram of *Geranium robertianum* hydroethanolic crude extract; (C) chromatogram of *Sambucus nigra* hydroethanolic crude extract; (D) chromatogram of *Viola odorata* hydroethanolic crude extract. Reference peaks were numbered based on their elution order: Ref 1 – Chlorogenic acid A 83, Rt=5.1 min.; Ref 2 – Violanthin, Rt=7.3 min.; Ref 3 – Kaempferol, Rt=12.3 min.

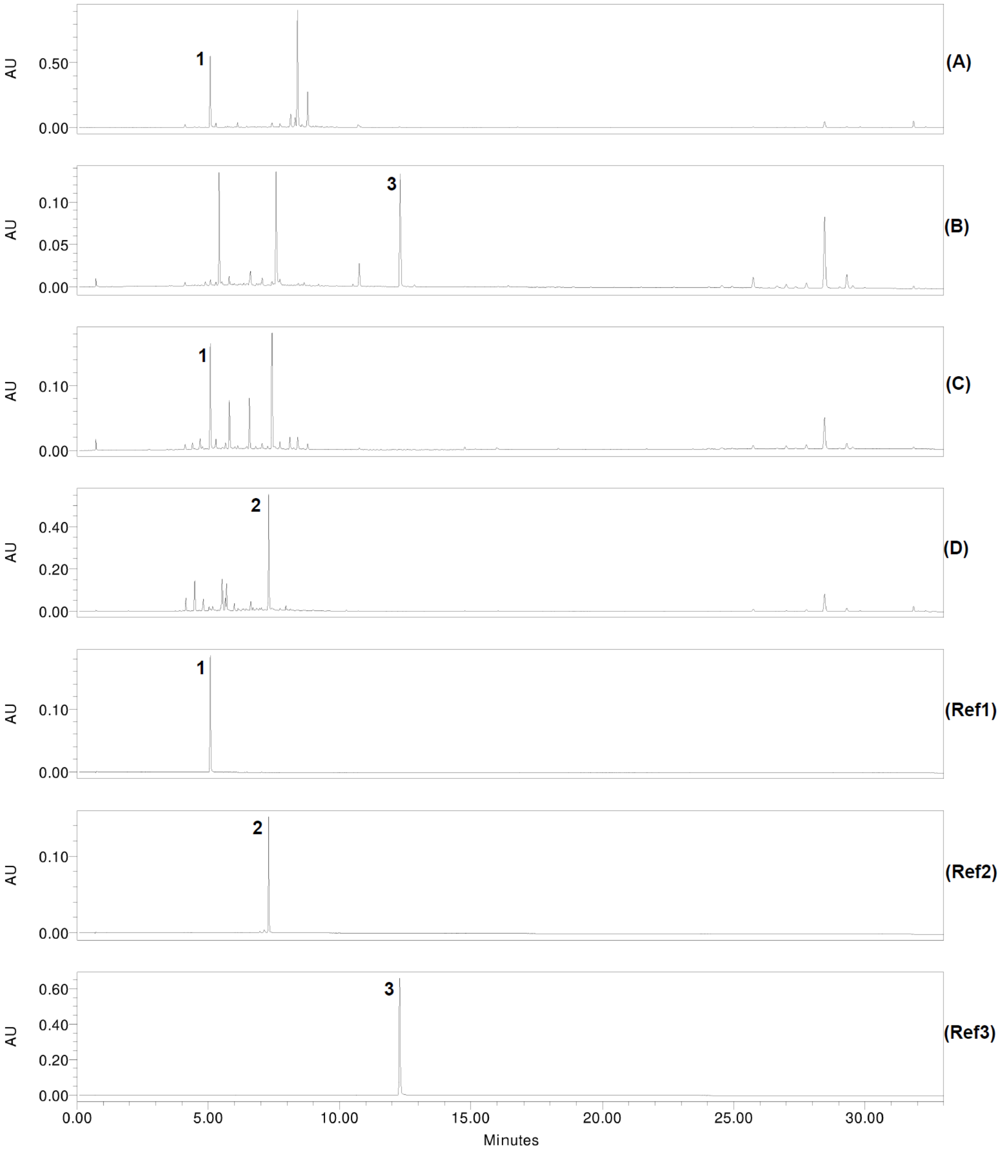

Figure S3. Stacked plots of UHPLC chromatograms at λ=350 nm. The four top chromatograms show the retained hydroethanolic crude extracts. The three chromatograms below show the reference markers. Reference peaks were numbered based on their elution order. Marker compounds of a reasonable height that were detected in the extract's chromatograms were numbered according to the reference peak. (A) chromatogram of *Artemisia vulgaris* hydroethanolic crude extract; (B) chromatogram of *Geranium robertianum* hydroethanolic crude extract; (C) chromatogram of *Sambucus nigra* hydroethanolic crude extract; (D) chromatogram of *Viola odorata* hydroethanolic crude extract. Reference peaks were numbered based on their elution order: Ref 1 – Chlorogenic acid A 83, Rt=5.1 min.; Ref 2 – Violanthin, Rt=7.3 min.; Ref 3 – Kaempferol, Rt=12.3 min.

	Chromatogram, UV 325 nm	Peak spectrum UV, 210-800 nm	Peak spectrum MS pos, 100-1250 m/z	Peak spectrum MS neg, 100-1250 m/z
**ViO**	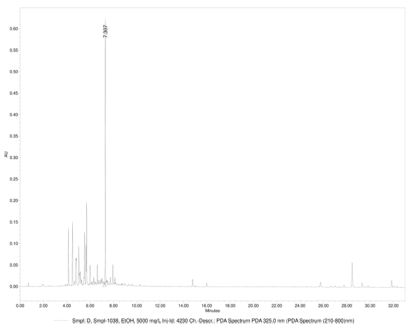	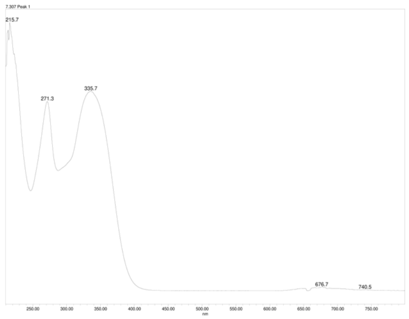	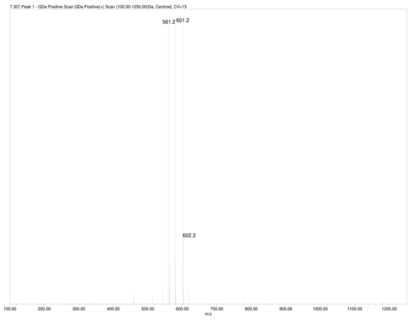	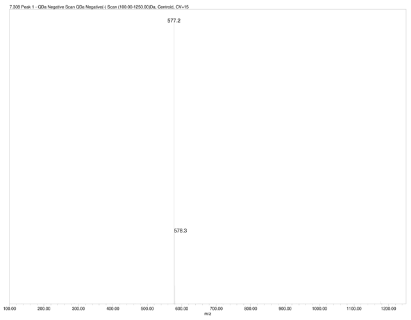
**Reference**	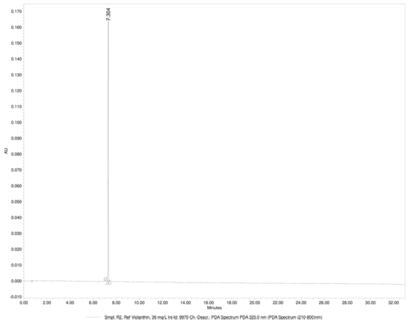	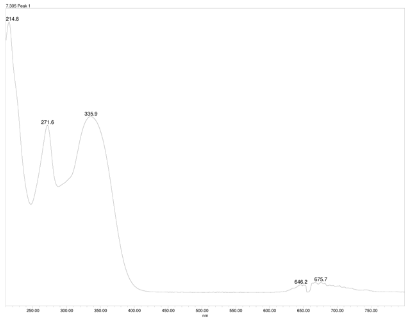	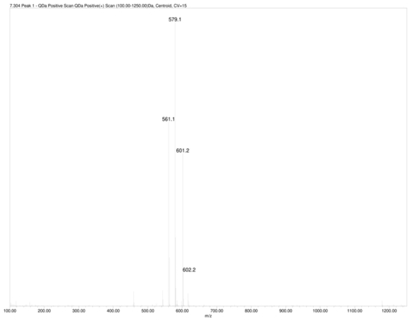	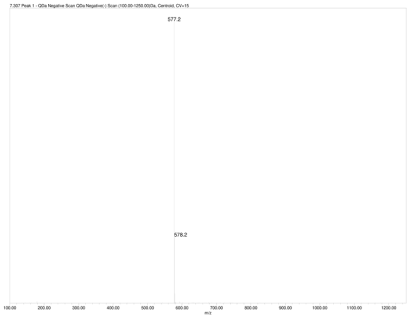

Figure S4. UV- and MS- spectra of *Viola odorata* ethyl acetate extract (ViO) and reference substance violanthin, Retention time R_t= 7,31 min.

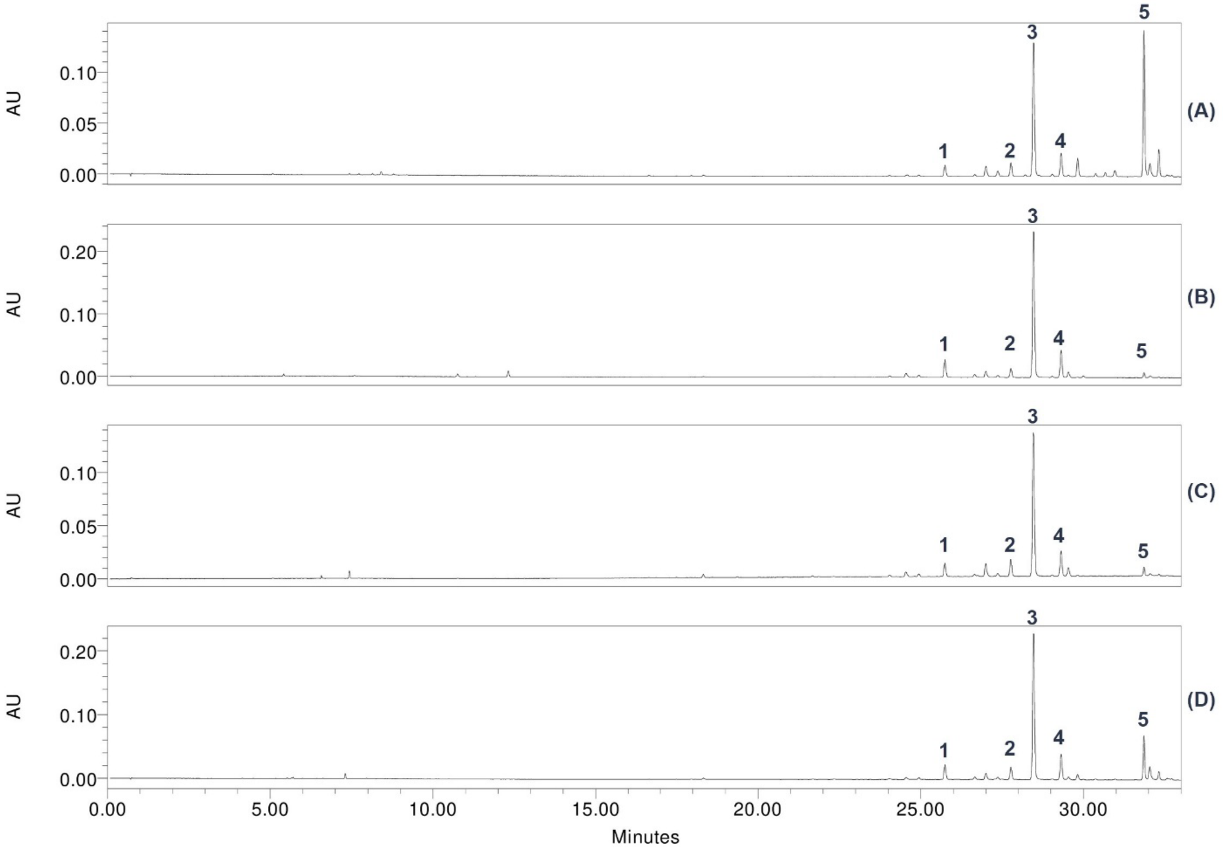

Figure S5. Stacked plots of UHPLC chromatograms at λ= 410 nm. The four chromatograms show the retained hydroethanolic crude extracts. The region of interest, extending from 25.5 min to 32 min., is composed of five common peaks (1-5). Peaks are numbered according to their elution order. (A) chromatogram of *Artemisia vulgaris* hydroethanolic crude extract; (B) chromatogram of *Geranium robertianum* hydroethanolic crude extract; (C) chromatogram of *Sambucus nigra* hydroethanolic crude extract; (D) chromatogram of *Viola odorata* hydroethanolic crude extract. Peak 3 (Rt=28.5 min.), which appears in all samples, is of pronounced height.

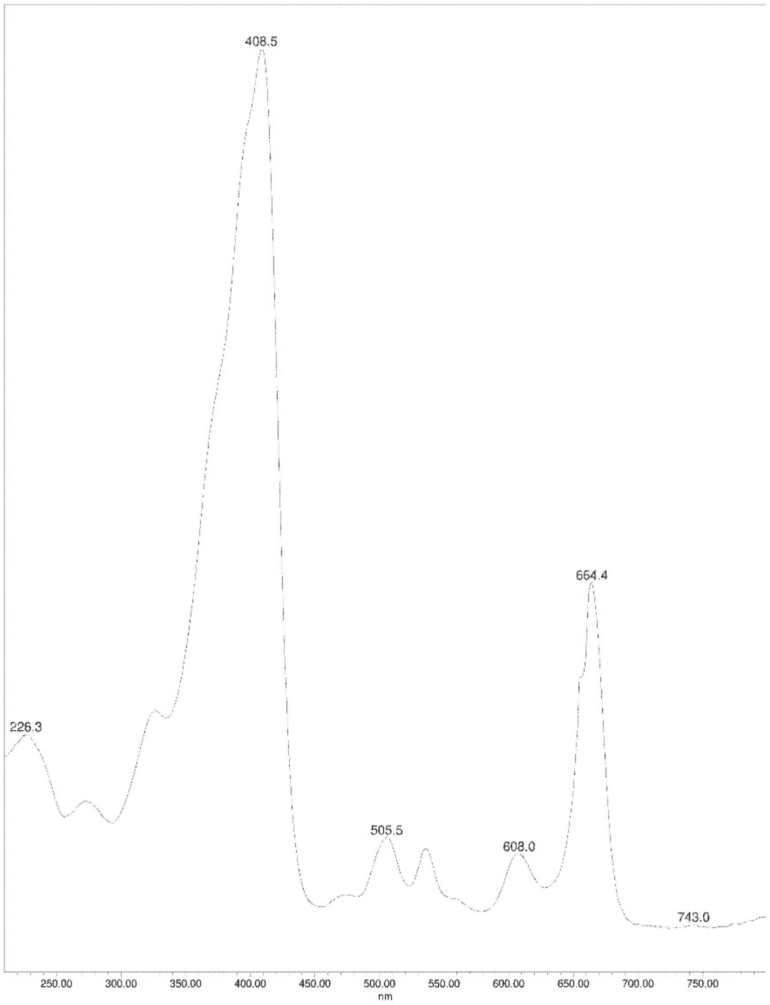

Figure S6. UV spectrum of the common peak 3 (Rt=28.5 min.) at λ= 410 nm. Two distinct absorption maxima are detected at 409 nm and 664 nm.
